# Automatic Prediction and Assessment of Treatment Response in Patients with Hodgkin’s Lymphoma Using a Whole-Body DW-MRI Based Approach

**DOI:** 10.3390/diagnostics10090702

**Published:** 2020-09-16

**Authors:** Valentina Brancato, Marco Aiello, Roberta Della Pepa, Luca Basso, Nunzia Garbino, Emanuele Nicolai, Marco Picardi, Marco Salvatore, Carlo Cavaliere

**Affiliations:** 1 IRCCS SDN (Istituto di Ricovero e Cura a Carattere Scientifico, SYNLAB istituto di Diagnostica Nucleare), 80143 Napoli, Italy; valentina.brancato@synlab.it (V.B.); luca.basso@synlab.it (L.B.); nunzia.garbino@synlab.it (N.G.); emanuele.nicolai@synlab.it (E.N.); direzionescientifica@sdn-napoli.it (M.S.); carlo.cavaliere@synlab.it (C.C.); 2 Department of Clinical Medicine and Surgery, Hematology Section, Federico II University of Naples, 80131 Naples, Italy; robertadellapepa@gmail.com (R.D.P.); marco.picardi@unina.it (M.P.)

**Keywords:** Hodgkin’s Lymphoma, whole-body DWI, automatic tool, segmentation, response to treatment, ROC analysis

## Abstract

The lack of validation and standardization represents the main drawback for a clear role of whole-body diffusion weighted imaging (WB-DWI) for prediction and assessment of treatment response in Hodgkin’s lymphoma (HL). We explored the reliability of an automatic approach based on the WB-DWI technique for prediction and assessment of response to treatment in patients with HL. The study included 20 HL patients, who had whole-body positron emission tomography (PET)/ magnetic resonance Imaging (MRI) performed before, during and after chemotherapy. Using the syngo.via MR Total Tumor Load tool, we automatically extracted values of diffusion volume (DV) and its associated histogram features by WB-DWI images, and evaluated their utility in predicting and assessing interim and end-of-treatment (EOT) response. The Mann–Whitney test followed by receiver operator characteristic (ROC) analysis was performed between features and their inter-time point percentage differences for patients having a complete or partial treatment response, revealing that several WB-DWI associated features allowed for prediction of interim response and both prediction and assessment of EOT response. Our proposed method offers huge advantages in terms of saving time and work, enabling clinicians to draw conclusions relating to HL treatment response in a fully automatic way, and encloses, also, all DWI advantages compared to PET/ computed tomography (CT).

## 1. Introduction

Hodgkin’s lymphoma (HL) is a relatively uncommon B-cell derived tumor, in which the unique cellular microenvironment is crucial for accurate diagnosis and pathobiology [1,2,3]. The role of diagnostic imaging provides important information for an accurate pretreatment evaluation and assessment of response to treatment, which are crucial steps for a good management of HL patients. In particular, the hybrid technique positron emission tomography (PET)/computed tomography (CT) with 18-fluorodeoxyglucose (^18^F-FDG) injection is considered the gold standard for HL management, from initial diagnosis to staging and assessment of response to treatment using the imaging-based Lugano classification [4,5,6,7].

Nevertheless, especially due to the harmful ionizing radiation dose involving both PET and CT modalities, there is an increasing interest towards integrated PET/MRI that combines the detailed morphological information provided by the radiation-free MRI with the functional information that characterizes PET images. The excellent soft tissue contrast displayed by MRI, together with the high PET sensitivity, allows to perform robust diagnostic evaluations. The role of MRI is strengthened by the possibility to include functional MRI techniques, such as diffusion weighted imaging (DWI), in the protocol. DWI is a noninvasive tool that allows to quantify the random motion of water molecules (diffusion), which becomes hampered in structures characterized by high cellularity, such as lymphoma lesions. PET/CT, whole-body DWI (WB-DWI), as well, provides both anatomical and functional information. The characteristic DWI parameter is the apparent diffusion coefficient (ADC), which allows for a quantitative evaluation of changes in tissue cellularity, providing a useful tool for diagnosis and assessment of response to treatment in tumors, in particular for lymphomas. Several studies investigate the power of DWI for HL and Non-Hodgkin’s (NHL) lymphoma diagnosis and assessment of treatment response compared to PET/CT, showing the potential role of DWI for these purposes [8].

Despite the continuous research and the promising results related to the usefulness of PET/MRI, especially when executed with DWI, the lack of validation and standardization represents the main drawback for a clear definition of the role of DWI in lymphoma diagnosis, staging, and response assessment. Moreover, it should be considered that, for a valid evaluation of response to treatment according to current guidelines, the readers need to have a deep experience and knowledge in the field of lymphomatous disease and their radiological evaluation [9]. Thus, we explore the possibility of having an automatic tool capable of providing support in lymphoma diagnosis.

MR Total Tumor Load stems from the promising results obtained by WB-DWI and ADC in multifocal disease, such as bone metastases and multiple myeloma, and the resulting need to dispose of an efficient tool able to evaluate this kind of lesions. This tool harnesses a threshold-based segmentation algorithm on whole-body diffusion-weighted images in order to identify regions of disease, and provides both the overall diffusion tumor volume and the histogram metrics of the corresponding computed ADC maps [10]. A high b-value image (acquired or computed) is used as input in order to maximize the contrast between lesions and healthy tissue. Several studies reported the advantages of using this tool especially in metastatic bone disease, but also in metastatic breast cancer and metastatic prostate cancer [10,11,12,13]. Another recent study (including three case studies) showed the benefits of the MR Total Tumor Load, not only in metastatic bone disease, but also in solid tumors. In particular, in one of these case studies concerning Hodgkin lymphoma in a 14-year-old girl, the reduction in tumor volume and the increase of low ADC values in ADC histogram between the pretreatment and the follow-up examination (after 2 months) are indicators of a good therapy response [14].

The purpose of this study is to evaluate the reliability of an automatic approach based on MR Total Tumor Load tool for WB-DWI technique relating to prediction and assessment of response to treatment in patients with HL, trying to detect the segmentation threshold, which is more capable of predicting and assessing response.

## 2. Materials and Methods

### 2.1. Patient Cohort

Twenty patients with histologically proven HL (11 men and 9 women), with a mean age of (35.7 ± 11.7) years were selected for this retrospective study. All subjects gave their informed consent for inclusion before they participated in the study. The study was conducted in accordance with the Declaration of Helsinki, and the protocol was approved by the Ethics Committee of the Istituto Nazionale Tumouri “Fondazione G. Pascale in 15 April 2020 (protocol number 3/20).

Inclusion criteria were patients being over the age of 18; histologic confirmation of HL at nodal biopsy; patients who underwent PET/CT followed by PET/MRI with WB-DWI at baseline before any treatment (T0), after two chemotherapy cycles (T1), and at the end-of-treatment (EOT) (T2), acquired from February 2016 to July 2018. All patients received doxorubicin (also known as Adriamycin), bleomycin, vinblastine, dacarbazine (ABVD) chemotherapy, and were asked to complete medical history questionnaires and sign informed consents to undergo hybrid PET-CT and PET-MRI investigations. Characteristics of included patients are shown in Table 1.

### 2.2. Acquisition Protocol

Data for all patients were acquired on both a PET-CT device and on a 3T hybrid PET-MRI system (Biograph mMR, Siemens, Erlangen, Germany) equipped with three 32-channel body coils, to cover the thorax, abdomen, and pelvis areas, and 12-channel phased array brain coils. Patients were asked to observe a fast of at least six hours. Sixty minutes after the 18-fluorodeoxyglucose (^18^F-FDG) injection by antecubital access, a PET-CT examination was performed from the brain vertex to the pelvis region. Then, patients underwent a whole-body PET-MRI protocol, which consisted of the following sequences: coronal T2 Turbo Inversion Recovery Magnitude (TIRM); an axial DWI sequence with b values of 50 and 800 s/mm^2^; axial and coronal T2 Half Fourier Acquisition Single Shot Turbo Spin Echo (HASTE), and an axial T1 Gradient Echo (GRE) in-out phase. The scan parameters are shown in Table 2. The attenuation correction is obtained from the segmentation (DIXON) into four classes, with predefined constant linear attenuation correction coefficients (LACs) for each class. The class denominations and the corresponding LACs were as follows: outer air (0 cm^−1^), lung (0.022 cm^−1^), fat tissue (0.085 cm^−1^), soft tissue (0.1 cm^−1^) [15]. Considering the PET data, the process of image reconstruction derived from an iterative algorithm called OSEM composed by three iterations on a matrix 172 × 172. Moreover, dividing data into 21 subsets analyzed cyclically, it was possible to control the noise within low absorption regions.

### 2.3. PET Response Evaluation

A radiologist and a nuclear medicine physician, respectively, with 7 and 10 years of experience, assessed, by consensus response, to treatment examining PET images on the basis of the visual Deauville 5-point scale (5-PS), according to the Lugano classification criteria in two sessions. In the first session, the interim response to treatment was assessed evaluating PET images acquired at interim and comparing them to those acquired at baseline; in the second session, the EOT response was assessed evaluating PET images acquired at the EOT and comparing them to those acquired at baseline. Patients were classified as having a complete metabolic response (CMR) in case of 5-PS score of 1, 2, or 3 in lymph nodal and extra lymphatic sites with or without a residual mass and no evident FDG-uptake in marrow. Partial metabolic response (PMR) in case of 5-PS score of 4 or 5 with reduced uptake compared with baseline, residual lesions of any size, and, relating to bone marrow, residual uptake higher than in normal marrow, but reduced compared with baseline. Stable metabolic disease (SMD), in case of 5-PS score of 4 or 5 with no evident change in FDG uptake, or progressive metabolic disease (PMD), in case of 5-PS score of 4 or 5 in any lesion with an increase in intensity of FDG uptake from baseline, and/or new FDG-avid foci, consistent with lymphoma, as well as new or recurrent FDG-avid sites in bone marrow [4,7]. For cases with a Deauville assigned score of 4 or 5 at T2, PMR, SMD, or PMD was defined considering also the interim PET scan.

### 2.4. WB-DWI Image Analysis and Data Extraction

**Figure 1:**
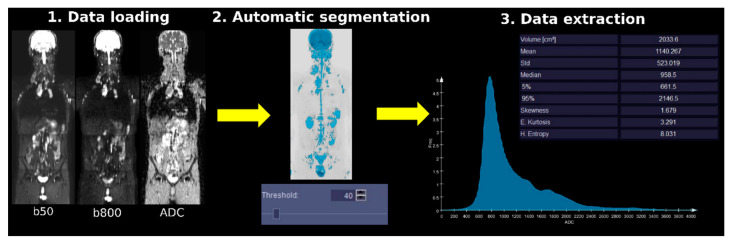
Whole-body diffusion weighted imaging (WB-DWI) image analysis and data extraction process using syngo.via Frontier MR Total Tumor Load software. Using the WB-DWI images as input (1. Data Loading step), b800 images were automatically segmented using setting a signal intensity threshold (e.g., 40%) at each WB-DWI acquisition time point (e.g., T0). No subsequent mask editing was made (2. Automatic segmentation). The overall mask volume (diffusion volume, DV) and the corresponding apparent diffusion coefficient (ADC) histogram metrics associated with the masked volume were extracted (3. Data extraction step). Yellow arrows link the three processing steps.

The analysis of WB-DWI images at each time point was performed using the syngo.via Frontier MR Total Tumor Load released research prototype v1.3.3 (Siemens Healthineers, Erlangen, Germany). For each patient, for each time point, the b800 images were used to automatically define threshold-based masks, using the threshold-based segmentation approach proposed by Blackedge et al. [16] and implemented in the syngo.via Frontier MR Total Tumor Load [10] (see Figure 1). Six segmentation threshold values were used, namely 5%, 10%, 20%, 40%, 60%, and 80%. No subsequent mask editing was made.

The overall mask volume (Diffusion Volume, DV) and the corresponding ADC histogram metrics associated with the masked volume were extracted. Specifically, extracted ADC histogram statistics were mean (ADCmean), standard deviation (ADCsd), median (ADCmd), 5% percentile (ADC5p), 95% percentile (ADC95p), skewness (ADCsk), excess kurtosis (ADCkurt), entropy (ADCentr). The following notation was used in this study to indicate the single feature:

$$f_{th\%}^{T},$$

where the superscript T indicates the time point at which WB-DWI images were acquired (T = 0, 1, 2), and the subscript th% indicates the segmentation threshold used (th = 5, 10, 20, 40, 60, 80). For each of the six thresholds, percentage changes in parameters during treatment from baseline and after treatment from baseline were calculated as follows:

(1) $$\Delta f_{th\%}^{0T}=\frac{\left(f_{th\%}^{T}-f_{th\%}^{0}\right)}{f_{th\%}^{0}}\times 100$$

where $f_{th\%}^{T}$ is the value of the feature at time point 1 or 2 and $f_{th\%}^{0}$ is the value of the feature at baseline. Percentage changes in parameters, after treatment from their values during treatment, were also calculated for each of the six thresholds as follows:

(2) $$\Delta f_{th\%}^{12}=\frac{\left(f_{th\%}^{2}-f_{th\%}^{1}\right)}{f_{th\%}^{1}}\times 100$$

where $f_{th\%}^{1}$ is the value of the feature at interim and $f_{th\%}^{2}$ is the value of the feature at the EOT.

### 2.5. Statistical Analysis

Values of each parameter were tested for normal distribution beforehand using a Kolmogorov–Smirnov test complemented by a graphical assessment for data normality using boxplots and Probability–Probability (P–P) plots, both overall and by subgroups identified, according to interim (T1) and EOT (T2) response. All normally distributed variables were summarized as mean (standard deviation), while those non-normally distributed as median and interquartile range (Q_1_; Q_3_).

In order to predict interim and EOT response, the *t*-test (in case of normally distributed parameters) or alternatively, the Mann–Whitney U test (in case of non-normally distributed parameters) on parameters at baseline, and also on parameters at interim for prediction of response at T2, was used to test the difference between CMR patients and each group of not CMR patients (PMR, SMD, PMD). For prediction of EOT response, percentage changes between features at T1 and T0 ($\Delta f_{th\%}^{01}$) was also evaluated. For parameters significant to t-test or Mann–Whitney test, receiver operator characteristic (ROC) curves were constructed and area under the curve (AUC) calculated to determine sensitivity and specificities and to find cut-off values that may be predictive of a poor response to treatment. The t-test or Mann–Whitney U test followed by ROC analysis for significant parameters was also performed on parameters at T1 and T2 as well as on percentage changes in parameters after treatment from baseline ($\Delta f_{th\%}^{02}$) and from interim ($\Delta f_{th\%}^{12}$) in order to evaluate their power in assessment of interim and EOT response.

Since none of the patients showed PMD or SMD at interim, and the population size for patients with PMD at the EOT was too small to perform statistical analysis (only two patients), we performed analysis for prediction of response to treatment only comparing patients with CMR and those with PMR. Specifically, we compared CMR and PMR patients at interim for prediction of interim response to treatment, and CMR and PMR patients at the EOT for prediction of EOT response.

All statistical analyses were performed using MATLAB (R2018a, MathWorks, Inc., Nettie, MA, USA). A *p*-value less than 0.05 was considered to indicate a statistically significant difference.

## 3. Results

### 3.1. Response to Therapy (Lugano Assessment)

**Figure 2:**
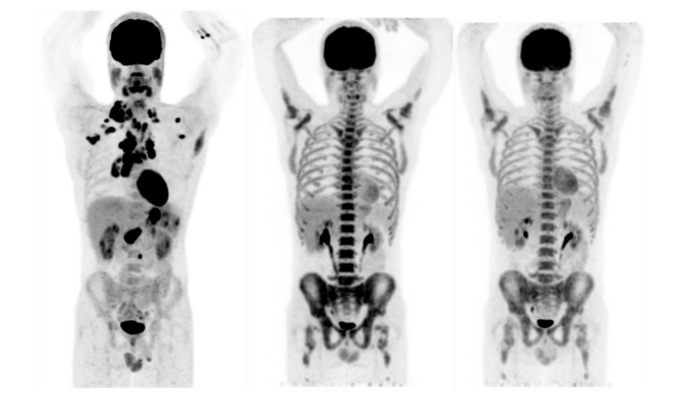
Maximum intensity projection (MIP) of positron emission tomography (PET) images at baseline, at interim and at the end-of-treatment (EOT) for a patient showing complete metabolic response (CMR), according to Lugano evaluation, both at T1 and T2.

Relating to the assessment of tumor response at interim, 14 patients (70%) showed CMR, while the remaining 6 patients (30%) had PMR. Relating to the assessment of tumor response at the EOT, 15 patients (75%) showed CMR, 3 (15%) had PMR, and 2 (10%) had PMD (see Table 3). In Figure 2, maximum intensity projection (MIP) of PET images at baseline, at interim and at the EOT for a patient showing CMR both at T1 and T2 are shown.

### 3.2. Image Analysis

**Figure 3:**
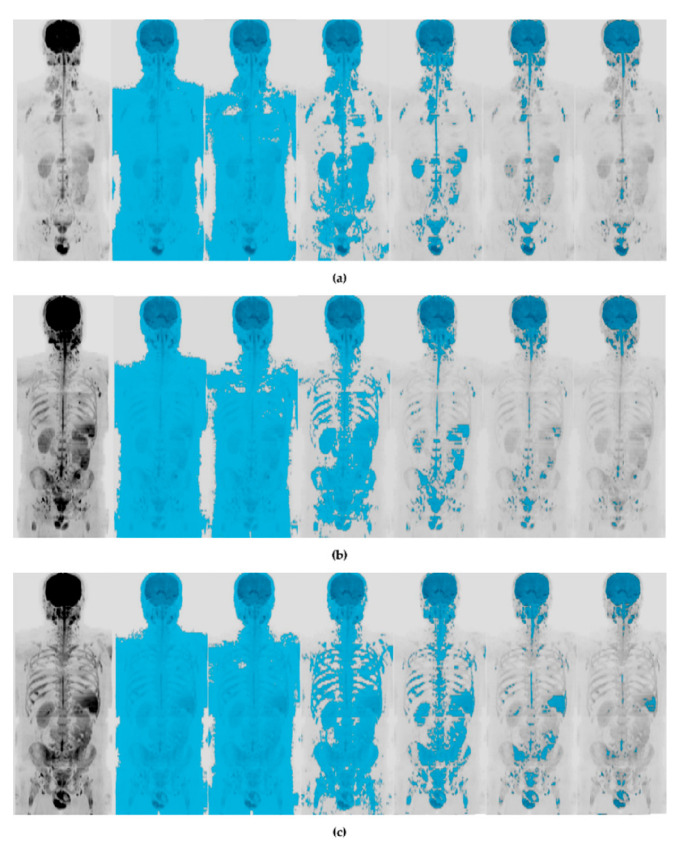
(**a**) Maximum intensity projection (MIP) of WB-DWI images at b800 at baseline (T0) and its relative masks at 5%, 10%, 20%, 40%, 60%, and 80%. (**b**) Maximum intensity projection (MIP) of whole-body DWI (WB-DWI) images at b800 at interim (T1) and its relative masks at 5%, 10%, 20%, 40%, 60%, and 80%. (**c**) Maximum intensity projection (MIP) of WB-DWI images at b800 at the EOT (T2) and its relative masks at 5%, 10%, 20%, 40%, 60%, and 80%. Images are from a patient showing complete metabolic response (according to Lugano evaluation) both at T1 and T2.

As shown in Figure 3, as the increase of the threshold, the tool became increasingly selective. Maximum intensity projection (MIP) of WB-DWI images acquired at b800 at baseline, at interim and at the EOT for a patient showing CMR both at T1 and T2 are also shown in Figure 3.

### 3.3. Prediction of Response to Treatment

The Kolmogorov–Smirnov test revealed that all baseline parameters, according to both T1 and T2 response, were non-Gaussian. Using Mann–Whitney U test, none of the baseline parameters was useful for prediction of interim response to therapy. Refer to Supplementary Table S1 for median values, interquartile ranges and associated *p*-values of all parameters, relating to prediction of interim response to treatment. Conversely, referring to prediction of EOT response, $DV_{40\%}^{0}$ and $DV_{60\%}^{0}$ were significantly higher in PMR patients than in CMR patients, while $ADC5p_{40\%}^{0}$ and $ADC95p_{20\%}^{1}$were significantly lower in PMR patients than in CMR patients. Refer to Table 4 for median values, *p*-values and associated ROC analysis statistics. See Supplementary Materials for median values, interquartile ranges and associated *p*-values of all parameters, relating to prediction of EOT response (Tables S2 and S3) and for boxplots and ROC curves of significant features (Figures S1–S4).

### 3.4. Assessment of Response to Treatment

Kolmogorov-Smirnov test revealed that all interim and EOT parameters, according to both T1 and T2 response, were non-Gaussian. Using Mann-Whitney U test for assessment of interim response to treatment, values for $ADCmean_{20\%}^{1}$, $ADCsd_{20\%}^{1}$, and $ADC95p_{}^{1}$ at 20% and 40% were significantly lower in PMR patients than in CMR patients. For thresholds from 5% to 40%, $\Delta ADCsd_{}^{01}$ was found to be significantly higher in CMR patients than in PMR patients. The same trend was observed for $\Delta ADC95p_{}^{01}$ at threshold of 5%, 20%, 40% and 60% and for $\Delta ADCentr_{}^{01}$ at thresholds from 5% to 40%. Conversely, values at interim of DV at 40% showed a completely opposite trend. In Table 5, median values and associated ROC analysis statistics for discrimination between CMR and PMR patients are reported. See Supplementary Tables S4 and S5 for median values, interquartile ranges, and associated *p*-values of all parameters relating to assessment of response to treatment at interim, and Figures S5–S22 for boxplots and ROC curves of significant features.

On the other hand, relating to assessment of EOT response, values at T2 of ADC mean and median at 5% threshold, $\Delta ADCsd_{5\%}^{02}$, $\Delta ADC5p_{60\%}^{02}$, and $\Delta ADCsd_{5\%}^{12}$ were significantly higher in PMR than in CMR patients. Conversely, $\Delta DV_{40\%}^{02}$ and $\Delta ADCsd_{60\%}^{02}$ revealed a completely opposite trend. Refer to Table 6 for median values, *p*-values, and associated ROC analysis statistics for discrimination between CMR and PMR patients. See Supplementary Materials for median values, interquartile ranges, and associated *p*-values of all parameters, relating to assessment of response to treatment at interim (Tables S6 and S7) and for boxplots and ROC curves of significant features (Figures S23–S29).

## 4. Discussion

The lack of validation and standardization represents the main drawback for a clear definition of the role of WB-DWI in lymphoma diagnosis, staging, and response assessment [17,18,19,20]. In the present preliminary study, we investigated a new WB-DWI-based approach for prediction and assessment of lymphoma response to treatment through the analysis of quantitative WB-DWI histogram features extracted from MR Total Tumor Load tool at six segmentation thresholds (5%, 10%, 20%, 40%, 60%, 80%) and using Lugano criteria applied on PET/CT images as reference standard.

Results of our study revealed the inability of all examined parameters to predict interim response. Concerning prediction of EOT response, DV at 40% and 60% was found to be significantly higher in PMR patients than in CMR patients. This could be related to the poorer response to treatment of PMR patients, that is normally associated with a higher diffusion tumor volume associated to DWI signal intensity [14,17]. Relating to ADC histogram variables, values at baseline of the 5th percentile of ADC at 40% and those of ADC skewness at 20% were respectively significantly lower and higher in PMR than in CMR patients and were found to be able to predict EOT response. Interim value of 95th percentile of ADC at 20% was also found to be useful for this purpose.

Interim value of DV at 40% was also useful for the assessment of interim response, as well as the following histogram related features: values at interim of ADC mean, standard deviation, and entropy at 20% threshold, and values of ADC 95th percentile at 5%, 20%, 40%, and 60%, were significantly lower in PMR than in CMR. The above-mentioned findings on ADC mean are consistent with the definition of PMR patients (which should have an overall lower ADC mean value than CMR patients) and in line with those obtained in the case study reported by Tsiflikas et al. [14] on a 14-year girl with HL, as well as in previous studies involving other tumor types and using MR Total Tumor Load Tool [4,10,12]. Percentage change between T1 and T0 in ADC standard deviation at 5%, 10%, 20%, and 40% was significantly lower in PMR patients than in CMR patients, and it was due to a completely opposite trend: in PMR patients there was a decrease in ADC standard deviation from T0 to T1, while, on the counter, in CMR patients this feature increased from T0 to T1. Same behavior was observed for percentage change between T1 and T0 in ADC 95th percentile at 20% and 40%, and in ADC entropy from 5% to 40% threshold.

Concerning the assessment of EOT response, mean and median values of ADC at the EOT were found to be significantly higher in PMR than in CMR patients. Significant results for assessment of EOT response were also achieved by percentage change between T2 and T0 in DV at 40% and in ADC 5th percentile at 60%, and by percentage change between T2 and T1 in ADC standard deviation at 5%.

Obtained results support the theory that DV and its related histogram-based ADC statistics could be useful in prediction and assessments of HL response to therapy, as observed in previous mentioned studies concerning different oncologic diseases and using MR Total Tumor Load Tool [10,12,14].

However, our results cannot be directly compared with any of these, since in our study, DV and its relative ADC histogram parameters were associated to a diffusion volume mask automatically generated by the tool, and so not manually segmented.

In fact, the innovation in our research is that we tried to draw conclusions skipping the mask editing step expected by the tool, which would require the intervention of an expert operator able to exclude normally hyperintense and not tumoral regions, and directly extract the features associated to the unrefined mask.

Our proposed method would surely offer huge advantages in terms of saving time and work, enabling also less expert operator to draw conclusions relating to lymphoma diagnosis. Moreover, being based on WB-DWI technique, it also encloses all DWI advantages compared to CT and PET, such as the absence of ionizing radiation, the fast acquisition of images and the no-requirement for contrast injection [21,22]. However, at the same time, it adds substantial limitations to our study. First of all, artifact regions and/or normal hyperintense regions were incorporated in the automatically segmented mask. For example, organs such as brain, kidneys, and spleen, are usually hyperintense. Moreover, due to its frequent activation due to chemotherapy, the bone marrow also appeared hyperintense, and this could justify an increase in diffusion volume during the chemotherapeutic treatment.

Limitations related to the gold standard are that the 5-PS and the subsequent Lugano assessment are a qualitative gold standard based on visual assessment, which results influenced by inter-observer variability due to the subjectivity of the interpretation [23]. We could have been accompanied this qualitative assessment with a semiquantitative one based on Standardized Uptake Value (SUV) values. In order to mitigate this limitation, Lugano assessment was performed in consensus between radiologist and nuclear medicine physician.

Furthermore, variations in time interval between the three image acquisitions and the different stage at T0 among patients may have influenced results.

Finally, the patient sample involved was small and unbalanced, and this might have adversely affected statistical results. Moreover, it should be considered that the retrospective study nature characterizing our study is supposed to have more bias and should be validated through prospective studies [24]. Despite this, the novelty of our method may provide a basis for future retrospective and prospective studies involving more participants. More informative results may be obtained if more patients could be investigated, also considering that, only three patients were found to have PMR response at EOT, making results on prediction and assessment of EOT response imprecise and unreliable. Moreover, only two patients were found to have PMD and no one SMD, not allowing us to perform comparisons among these categories.

It could be interesting to evaluate PET images using the same method, and compare them with WB-DWI images, both in terms of histogram parameters and relating to masked total volume, respectively, total diffusion volume (DV) in WB-DWI and total metabolic volume in PET), and investigate how unrefined diffusion volume masks extracted by the tool are related with SUV values associated with PET of PET/MRI. Following this line, it would be attractive to investigate the utility of the unrefined total metabolic volume automatically extracted by the tool for prediction and assessment of response to treatment in lymphoma patients, as done by Cottereau et al. [25,26] considering the total metabolic tumor volume (TMTV). Moreover, it would be interesting to integrate imaging information with those arising from lymph node biopsy (which is the gold standard for diagnosing lymphoma) and also from liquid biopsy markers, in order to choose a tailored treatment strategy for each HL patient and better evaluate treatment efficacy [27,28,29].

## 5. Conclusions

In conclusion, in this preliminary study, we found that several WB-DWI associated features allowed for prediction of interim response and both prediction and assessment of EOT response of patients with HL. However, the novelty of our method of feature extraction, with its related restrictions, and the lack of a defined and standardized role of DWI for the management of HL, pave the way for further studies involving larger groups of patients, which are essential to investigate the effective impact of our method and validate obtained results.

## Figures and Tables

**Table 1:** Characteristics of study population.

Variable	Value
No. of patients (*n*)	20
Age (y)	
Mean ± SD	35.7 ± 11.7
Range	19–63
Gender (*n* (%))	
Male	11 (55)
Female	9 (45)
HL Subtype (WHO classification) (*n* (%))	
Nodular sclerosis	13 (65)
Mixed cellularity	4 (20)
Lymphocyte rich	2 (10)
Lymphocyte depleted	1 (5)
B symptoms (*n* (%))	
Fever	14 (70)
Sweats	9 (45)
Weight loss ≥ 10%	6 (30)
Histology (*n* (%))	
Stage	
I	2 (10)
II	11 (55)
III	4 (20)
IV	3 (15)
Erythrocyte sedimentation rate ≥ 50 mm	2 (10)

Abbreviations: SD = Standard Deviation; HL = Hodgkin’s Lymphoma; WHO = World Health Organization.

**Table 2:** Scan parameters for whole-body MRI-DWI.

Sequence	Orientation	TR (ms)	TE (ms)	ST (mm)	TI (mm)	b-Values (s/mm^2^)
T2 TIRM	Coronal	4500	84	5	220	
DWI	Axial		78	6	220	50,800
T2 HASTE	Axial and Coronal	1400	89	6		
T1 GRE	Axial	94	2.46	5		

Abbreviations: T2 TIRM = Turbo Inversion Recovery Magnitude; DWI = Diffusion-Weighted Imaging; T2 HASTE = Half Fourier Acquisition Single Shot Turbo Spin Echo; T1 GRE = Gradient Echo; TR: = Repetition Time; TE = Echo Time; ST = Slice Thickness; TI = Inversion Time.

**Table 3:** Results of response to therapy according to Lugano assessment. Reported data are number of patients and percentages are in parenthesis.

Lugano Assessment	Response to Therapy at Interim (T1) (*n* (%))	Response to Therapy at the EOT (T2) (*n* (%))
CMR	14 (70)	15 (75)
PMR	6 (30)	3 (15)
SMD	0 (0)	0 (0)
PMD	0 (0)	2 (10)

Abbreviations: EOT = end of treatment; CMR = complete metabolic response; PMR = partial metabolic response; SMD = stable metabolic disease; PMD = progressive metabolic disease.

**Table 4:** Significant results for prediction of end-of-treatment response.

Parameters ^a^	Median (IQR)—CMR	Median (IQR)—PMR	*p*	AUC	T	Sen (%)	Spec (%)	Acc (%)
$DV_{40\%}^{0}$ (cm^3^)	2033.6 (1907.5–2087.75)	2446.1 (2439.88–2542.55)	0.017	0.93	2342.15	100	93	94
$DV_{60\%}^{0}$ (cm^3^)	1525.7 (1440.48–1556.23)	1667.4 (1626.23–1733.4)	0.01	0.96	1604.6	100	93	94
$ADC5p_{40\%}^{0}$ (×10^−6^ mm^2^/s)	634.5 (607.5–634.5)	580.5 (540–583.88)	0.037	0.89	596.25	80	100	83
$ADC95p_{20\%}^{1}$ (×10^−6^ mm^2^/s)	2686.5 (2416.5–2902.5)	2335.5 (2254.5–2376)	0.032	0.91	2403	85	100	88

^a^ superscripts indicate WB-DWI acquisition time point; subscripts indicate segmentation threshold. IQR = Interquartile Range; CMR = Complete Metabolic Response; PMR = Partial Metabolic Response; *p* = *p*-value; AUC = Area under the ROC Curve; T = Optimal Threshold; Sen = Sensitivity; Spec = Specificity; Acc = Accuracy; DV = Diffusion Volume; ADC = Apparent Diffusion Coefficient; ADC5p = ADC 5% percentile; ADC95p = ADC 95% percentile. Units of measurement for each parameter is shown in square brackets.

**Table 5:** Significant results for assessment of interim response to treatment.

Parameters ^a^	Median (IQR)—CMR	Median (IQR)—PMR	*p*	AUC	T	Sen (%)	Spec (%)	Acc (%)
$DV_{40\%}^{1}$ (cm^3^)	1916.35 (1846.85–2084.55)	2311.45 (2235.5–2446.7)	0.005	0.90	2116.5	100	83	89
$ADCmean_{20\%}^{1}$ (×10^−6^ mm^2^/s)	1311.58 (1252.74–1356.32)	1221.34 (1111.06–1240.01)	0.009	0.88	1243.85	83	83	83
$ADCsd_{20\%}^{1}$ (×10^−6^ mm^2^/s)	695.49 (644.57–737.93)	609.96 (577.22–635.21)	0.009	0.88	656.93	75	100	83
$ADC95p_{20\%}^{1}$ (×10^−6^ mm^2^/s)	2686.5 (2497.5–2902.5)	2281.5 (2227.5–2389.5)	0.001	0.93	2403	92	100	94
$ADC95p_{40\%}^{1}$ (×10^−6^ mm^2^/s)	2646 (2349–2796.75)	2214 (2093–2335.5)	0.013	0.86	2362.5	75	83	78
$ADCentr_{20\%}^{1}$ (×10^−6^ mm^2^/s)	8.49 (8.4–8.54)	8.37 (8.3–8.47)	0.024	0.83	8.48	67	100	78
$\Delta ADCsd_{5\%}^{01}$ (%)	4.85 (1.87–5.66)	−4.79 (−9.43 to −2.67)	0.006	0.92	0.81	83	100	88
$\Delta ADCsd_{10\%}^{01}$ (%)	2.69 (−0.08 to 5.12)	−7.38 (−10.08 to −0.04)	0.036	0.83	0.1	75	80	76
$\Delta ADCsd_{20\%}^{01}$ (%)	8.47 (−2.99 to 12.54)	−10.37 (−14.92 to −5.93)	0.013	0.88	−6.79	83	80	82
$\Delta ADCsd_{40\%}^{01}$ (%)	7.76 (−0.09 to 12)	−9.52 (−21.81 to −2.84)	0.019	0.87	−2.05	83	100	88
$\Delta ADC95p_{5\%}^{01}$ (%)	0.44 (−1.41 to 3.49)	−2.72 (−8.86 to −2.36)	0.045	0.82	−2.09	0.83	0.8	0.82
$\Delta ADC95p_{20\%}^{01}$ (%)	2.8 (−5.56 to 6.43)	−18.43 (−20.71 to −6.46)	0.013	0.88	−5.52	75	80	76
$\Delta ADC95p_{40\%}^{01}$ (%)	4.65 (−3.64 to 13.14)	−10.33 (−24.45 to −1.82)	0.02	0.86	−1.21	75	80	76
$\Delta ADC95p_{60\%}^{01}$ (%)	1.95 (−4.34 to 6.81)	−3.85 (−15.68 to 0.31)	0.048	0.82	1.28	0.75	1	0.82
$\Delta ADCentr_{5\%}^{01}$ (%)	0.32 (−0.04 to 0.56)	−0.53 (−1.24 to −0.19)	0.013	0.88	−0.27	92	8	88
$\Delta ADCentr_{10\%}^{01}$ (%)	0.27 (0.16–0.56)	−0.51 (−1.16 to −0.03)	0.019	0.87	0.02	83	80	82
$\Delta ADCentr_{20\%}^{01}$ (%)	0.71 (−0.27 to 1.21)	–0.83 (−2.16 to −0.39)	0.009	0.90	−0.03	75	100	82
$\Delta ADCentr_{40\%}^{01}$ (%)	0.88 (−0.18 to 1.36)	–1.42 (−1.65 to −0.92)	0.013	0.88	−1.15	92	80	88

^a^ superscripts indicate one or two (in case of percentage differences) WB-DWI acquisition time points; subscripts indicate segmentation threshold. IQR = Interquartile Range; CMR = Complete Metabolic Response; PMR = Partial Metabolic Response; *p* = *p*-value; AUC = Area Under the ROC Curve; T = Optimal Threshold; Sen = Sensitivity; Spec = Specificity; DV = Diffusion Volume; ADC = Apparent Diffusion Coefficient; ADCmean = ADC mean; ADCsd = ADC standard deviation; ADC95p = ADC 95% percentile; ADCentr = ADC entropy; ∆ADC = percentage change of ADC between two time points; ∆ADCsd = percentage change in ADC standard deviation; ∆ADC95p = percentage change in ADC 95% percentile; ∆ADCentr = percentage change in ADC entropy.

**Table 6:** Significant results for assessment of end-of-treatment response.

Parameters ^a^	Median (IQR)—CMR	Median (IQR)—PMR	*p*	AUC	T	Sen (%)	Spec (%)	Acc (%)
$ADCmean_{5\%}^{2}$ (×10^−6^ mm^2^/s)	1103.54 (1061.37–1159.35)	1211.08 (1180.53–1227.21)	0.014	0.95	1169.09	100	85	88
$ADCmd_{5\%}^{2}$ (×10^−6^ mm^2^/s)	1066.5 (1034.44–1174.5)	1228.5 (1208.25–1289.25)	0.01	0.96	1188	100	92	94
$\Delta DV_{40\%}^{02}$ (%)	15.04 (4.49–34.57)	−10.05 (−22.72 to −1.69)	0.025	0.92	4.06	77	100	81
$\Delta ADCsd_{5\%}^{02}$ (%)	−2.09 (−4.8 to 2.38)	4.6 (2.99–4.91)	0.039	0.9	2.36	100	77	81
$\Delta ADCsd_{60\%}^{02}$ (%)	−0.84 (−5.82 to 4.66)	–23.12 (−28.21 to −10.25)	0.025	0.92	−5.79	77	100	81
$\Delta ADC5p_{60\%}^{02}$ (%)	−4.26 (−6.69 to 0.91)	4.26 (4.26–7.26)	0.014	0.95	4.17	100	92	94
$\Delta ADCsd_{5\%}^{12}$ (%)	−1.79 (−4.5 to 1.22)	6.35 (5.09–10.09)	0.017	0.94	4.21	100	92	93

^a^ superscripts indicate one or two (in case of percentage differences) DWI acquisition time points; subscripts indicate segmentation threshold. IQR = Interquartile Range; CMR = Complete Metabolic Response; PMR = Partial Metabolic Response; *p* = *p*-value; AUC = Area Under the ROC Curve; T = Optimal Threshold; Sen = Sensitivity; Spec = Specificity; DV = Diffusion Volume; ADC = Apparent Diffusion Coefficient; ADCmean = ADC mean; ADCmd = ADC median; ∆ADCsd = percentage change in ADC standard deviation; ∆ADC5p = percentage change in ADC 5% percentile.

## Supplementary Materials

The following are available online at https://www.mdpi.com/2075-4418/10/9/702/s1, Tables S1–S7, Figures S1–S29.
