# Supplementary material for: Automatic Prediction and Assessment of Treatment Response in Patients with Hodgkin’s Lymphoma Using a Whole-Body DW-MRI Based Approach
Source: Diagnostics (Basel). 2020 Sep 16;10(9):702. doi: 10.3390/diagnostics10090702 (PMC7555579; doi:10.3390/diagnostics10090702)
Supplement: Supplementary file 1 [file diagnostics-10-00702-s001.pdf]

diagnostics

Automatic Prediction and Assessment of Treatment Response in Patients with Hodgkin's Lymphoma Using a Whole-Body DW-MRI Based Approach

Valentina Brancato [1], Marco Aiello [1,*], Roberta Della Pepa [2], Luca Basso [1], Nunzia Garbino [1], Emanuele Nicolai [1], Marco Picardi [2], Marco Salvatore [1] and Carlo Cavaliere [1]

S1. Median values, Interquartile ranges and results of Mann-Whitney U test for all parameters.

Prediction of intermediate response to treatment

Table S1. Median values, Interquartile ranges (IQR) and results of Mann-Whitney U test of parameters for prediction of intermediate response to treatment.

T0 PARAMETERS TO PREDICT T1 RESPONSE			
**Parameters**	**Median (IQR) - CMR**	**Median (IQR) - PMR**	**p**
DV [cm³]			
$DV^0_{5\%}$	34662.02 (31131.1 – 41000.9)	29272.2 (26798.6 – 35252.3)	NS
$DV^0_{10\%}$	15985.19 (14931.4 – 17981.2)	14665.5 (14091.25 – 17204.73)	NS
$DV^0_{20\%}$	3997.88 (3468.4 – 4318.3)	4312.5 (4154.73 – 5004.08)	NS
$DV^0_{40\%}$	2041.35 (1900.6 – 2095.1)	2437.8 (1986.85 – 2478.25)	NS
$DV^0_{60\%}$	1518.35 (1468 – 1550.6)	1612.5 (1437.45 – 1689.4)	NS
$DV^0_{80\%}$	1165.95 (1117.3 – 1201.3)	1171.4 (1116.08 – 1220.88)	NS
ADCmean [×10⁻⁶ mm²/s]			
$ADCmean^0_{5\%}$	1106.55 (1031.21 – 1152.06)	1199.33 (1101.45 – 1272.92)	NS
$ADCmean^0_{10\%}$	1197.74 (1115.69 – 1278.13)	1235.69 (1162.7 – 1369.51)	NS
$ADCmean^0_{20\%}$	1329.52 (1303.88 – 1397.24)	1331.71 (1279.68 – 1459.43)	NS
$ADCmean^0_{40\%}$	1218.25 (1168.62 – 1287.03)	1284.87 (1167.22 – 1362.5)	NS
$ADCmean^0_{60\%}$	1132.7 (1090.44 – 1161.46)	1129.94 (1046.01 – 1309.29)	NS
$ADCmean^0_{80\%}$	1074.36 (1016.08 – 1132.19)	1091.1 (1012.39 – 1239.11)	NS
ADCsd [×10⁻⁶ mm²/s]			
$ADCsd^0_{5\%}$	559.97 (535.35 – 590)	590.42 (563.02 – 622.25)	NS
$ADCsd^0_{10\%}$	599.14 (576.61 – 609.58)	606.16 (587.56 – 626.52)	NS
$ADCsd^0_{20\%}$	642.32 (612.35 – 658.94)	693.06 (616.82 – 731.63)	NS
$ADCsd^0_{40\%}$	608.23 (569.64 – 622.89)	741.63 (533.25 – 762.21)	NS
$ADCsd^0_{60\%}$	544.77 (494.86 – 594.73)	600.91 (448.07 – 689.58)	NS
$ADCsd^0_{80\%}$	486.47 (442.18 – 523.97)	481.23 (389.58 – 623.44)	NS
ADCmd [×10⁻⁶ mm²/s]			
$ADCmd^0_{5\%}$	1107 (1039.5 – 1174.5)	1228.5 (1093.5 – 1269)	NS
$ADCmd^0_{10\%}$	1215 (1147.5 – 1282.5)	1255.5 (1174.5 – 1370.25)	NS
$ADCmd^0_{20\%}$	1174.5 (1147.5 – 1228.5)	1147.5 (1140.75 – 1329.75)	NS
$ADCmd^0_{40\%}$	999 (958.5 – 1066.5)	985.5 (976.38 – 1093.5)	NS
$ADCmd^0_{60\%}$	923 (887.5 – 975)	912.5 (894.63 – 1007.5)	NS
$ADCmd^0_{80\%}$	931.77 (891 – 972)	924 (900.38 – 1008)	NS
ADC5p [×10⁻⁶ mm²/s]			
$ADC5p^0_{5\%}$	256.5 (175.5 – 310.5)	337.5 (243 – 351)	NS
$ADC5p^0_{10\%}$	285.58 (202.5 – 364.5)	418.5 (243 – 459)	NS
$ADC5p^0_{20\%}$	621 (580.5 – 661.5)	553.5 (526.5 – 621)	NS

$ADC5p_{40\%}^{0}$	634.5 (607.5 – 634.5)	580.5 (567 – 604.13)	NS
$ADC5p_{60\%}^{0}$	629.54 (611 – 637.5)	587.5 (573.88 – 624)	NS
$ADC5p_{80\%}^{0}$	617.71 (609.5 – 637.5)	588 (580.88 – 642)	NS
***ADC95p* [×10⁻⁶ *mm²/s*]**			
$ADC95p_{5\%}^{0}$	1997.73 (1950.5 – 2038.5)	2011.5 (1984.5 – 2254.5)	NS
$ADC95p_{10\%}^{0}$	2187 (2092.5 – 2362.5)	2200.5 (2112.75 – 2443.5)	NS
$ADC95p_{20\%}^{0}$	2578.5 (2443.5 – 2659.5)	2929.5 (2382.75 – 2997)	NS
$ADC95p_{40\%}^{0}$	2416.5 (2389.5 – 2524.5)	2875.5 (2166.38 – 3064.5)	NS
$ADC95p_{60\%}^{0}$	2311.08 (2173.5 – 2431)	2512.5 (2008.13 – 2710.5)	NS
$ADC95p_{80\%}^{0}$	2153.48 (2004 – 2311.5)	2100 (1841.63 – 2550)	NS
***ADCsk* [$\times 10^{-6}$ *mm²/s*]**			
$ADCsk_{5\%}^{0}$	0.7 (0.65 – 0.77)	0.7 (0.64 – 0.77)	NS
$ADCsk_{10\%}^{0}$	0.79 (0.68 – 0.94)	0.78 (0.66 – 1.03)	NS
$ADCsk_{20\%}^{0}$	1.04 (0.96 – 1.11)	1.12 (0.91 – 1.27)	NS
$ADCsk_{40\%}^{0}$	1.3 (1.1 – 1.62)	1.39 (1.08 – 1.49)	NS
$ADCsk_{60\%}^{0}$	1.55 (1.43 – 1.83)	1.75 (1.14 – 1.83)	NS
$ADCsk_{80\%}^{0}$	1.51 (1.41 – 1.78)	1.55 (1.28 – 1.72)	NS
***ADCkurt* [$\times 10^{-6}$ *mm²/s*]**			
$ADCkurt_{5\%}^{0}$	1.61 (1.52 – 1.79)	1.7 (1.31 – 2.2)	NS
$ADCkurt_{10\%}^{0}$	1.94 (1.55 – 2.1)	2.17 (1.56 – 2.56)	NS
$ADCkurt_{20\%}^{0}$	1.07 (0.76 – 1.2)	1.47 (0.97 – 1.53)	NS
$ADCkurt_{40\%}^{0}$	1.19 (0.48 – 2.6)	1.59 (0.3 – 2.08)	NS
$ADCkurt_{60\%}^{0}$	2.07 (1.47 – 3.54)	2.88 (0.23 – 3.9)	NS
$ADCkurt_{80\%}^{0}$	2.28 (1.58 – 3.67)	2.6 (0.88 – 3.76)	NS
***ADCentr* [$\times 10^{-6}$ *mm²/s*]**			
$ADCentr_{5\%}^{0}$	8.19 (8.16 – 8.28)	8.29 (8.24 – 8.37)	NS
$ADCentr_{10\%}^{0}$	8.33 (8.26 – 8.37)	8.36 (8.32 – 8.42)	NS
$ADCentr_{20\%}^{0}$	8.41 (8.33 – 8.48)	8.48 (8.39 – 8.52)	NS
$ADCentr_{40\%}^{0}$	8.2 (8.12 – 8.32)	8.34 (8.09 – 8.44)	NS
$ADCentr_{60\%}^{0}$	8.03 (7.99 – 8.11)	8.12 (7.89 – 8.28)	NS
$ADCentr_{80\%}^{0}$	7.98 (7.85 – 8.06)	8.03 (7.81 – 8.16)	NS

Prediction of final response to treatment

Table S2. Median values, Interquartile ranges (IQR) and results of Mann-Whitney U test of parameters for prediction of final response to treatment (evaluating T0 and T1 parameters and using Lugano on T2 images).

T0 PARAMETERS TO PREDICT T2 RESPONSE			
**Parameters**	**Median (IQR) - CMR**	**Median (IQR) - PMR**	**p**
DV [cm³]			
$DV^0_{5\%}$	35853.87 (31325.67 – 40858.9)	29272.2 (27780.6 – 33138.75)	NS
$DV^0_{10\%}$	16118.86 (15010.38 – 18098.13)	14334.4 (13604.95 – 15891.93)	NS
$DV^0_{20\%}$	4049.3 (3508.4 – 4457.05)	4312.5 (4256.33 – 4664.55)	NS
$DV^0_{40\%}$	2033.6 (1907.5 – 2087.75)	2446.1 (2439.88 – 2542.55)	0.017
$DV^0_{60\%}$	1525.7 (1440.48 – 1556.23)	1667.4 (1626.23 – 1733.4)	0.01
$DV^0_{80\%}$	1148.5 (1081.38 – 1200.57)	1196.9 (1177.78 – 1268.82)	
ADCmean [×10⁻⁶ mm²/s]			
$ADCmean^0_{5\%}$	1119.73 (1041.74 – 1177.68)	1199.33 (1130.88 – 1217.97)	NS
$ADCmean^0_{10\%}$	1213.64 (1122.97 – 1283.97)	1235.69 (1188.3 – 1285.53)	NS
$ADCmean^0_{20\%}$	1331.53 (1304.05 – 1456.34)	1331.71 (1270.34 – 1374.75)	NS
$ADCmean^0_{40\%}$	1246.56 (1170.93 – 1321.14)	1284.87 (1168.21 – 1309.04)	NS
$ADCmean^0_{60\%}$	1140.98 (1098.61 – 1178.36)	1129.94 (1059.72 – 1257.36)	NS
$ADCmean^0_{80\%}$	1089.34 (1019.14 – 1151.99)	1091.1 (1037.62 – 1175.18)	NS
ADCsd [×10⁻⁶ mm²/s]			
$ADCsd^0_{5\%}$	570.05 (537.53 – 614.72)	577.31 (534.44 – 587.87)	NS
$ADCsd^0_{10\%}$	605.84 (576.74 – 621.12)	605.72 (551.23 – 606.05)	NS
$ADCsd^0_{20\%}$	648.64 (614.39 – 667)	726.47 (615.38 – 741.96)	NS
$ADCsd^0_{40\%}$	615.7 (572.15 – 644.26)	741.63 (579.8 – 782.24)	NS
$ADCsd^0_{60\%}$	554.46 (495.99 – 603.74)	600.91 (486.61 – 736.02)	NS
$ADCsd^0_{80\%}$	491.07 (447.13 – 545.1)	481.23 (415.33 – 625.87)	NS
ADCmd [×10⁻⁶ mm²/s]			
$ADCmd^0_{5\%}$	1120.5 (1046.25 – 1174.5)	1228.5 (1127.25 – 1228.5)	NS
$ADCmd^0_{10\%}$	1228.5 (1161 – 1282.5)	1255.5 (1194.75 – 1296)	NS
$ADCmd^0_{20\%}$	1174.5 (1147.5 – 1248.75)	1147.5 (1127.25 – 1208.25)	NS
$ADCmd^0_{40\%}$	1012.5 (958.5 – 1066.5)	985.5 (958.13 – 1005.75)	NS
$ADCmd^0_{60\%}$	923 (887.5 – 982.88)	912.5 (893.75 – 939.88)	NS
$ADCmd^0_{80\%}$	948 (895.38 – 972)	924 (912.38 – 924)	NS
ADC5p [×10⁻⁶ mm²/s]			
$ADC5p^0_{5\%}$	262.29 (182.25 – 330.75)	337.5 (276.75 – 378)	NS
$ADC5p^0_{10\%}$	302.79 (209.25 – 364.5)	418.5 (297 – 438.75)	NS
$ADC5p^0_{20\%}$	634.5 (580.5 – 661.5)	553.5 (533.25 – 594)	NS
$ADC5p^0_{40\%}$	634.5 (607.5 – 634.5)	580.5 (540 – 583.88)	0.037
$ADC5p^0_{60\%}$	618.04 (608.38 – 637.38)	587.5 (587.5 – 605.13)	NS

$ADC5p^{0}_{80\%}$	612 (609.5 – 637.13)	588 (586.88 – 624)	NS
ADC95p [×10⁻⁶ mm²/s]			
$ADC95p^{0}_{5\%}$	2011.5 (1952.25 – 2063.45)	2011.5 (1991.25 – 2092.5)	NS
$ADC95p^{0}_{10\%}$	2200.5 (2092.5 – 2484)	2200.5 (2139.75 – 2301.75)	NS
$ADC95p^{0}_{20\%}$	2605.5 (2457 – 2822.46)	2983.5 (2457 – 3024)	NS
$ADC95p^{0}_{40\%}$	2416.5 (2396.25 – 2585.25)	3064.5 (2374.88 – 3064.5)	NS
$ADC95p^{0}_{60\%}$	2337.5 (2185.88 – 2435.88)	2512.5 (2118.75 – 2880.38)	NS
$ADC95p^{0}_{80\%}$	2172 (2028 – 2314.88)	2100 (1913.63 – 2694)	NS
ADCsk [×10⁻⁶ mm²/s]			
$ADCsk^{0}_{5\%}$	0.7 (0.64 – 0.77)	0.74 (0.69 – 0.84)	NS
$ADCsk^{0}_{10\%}$	0.75 (0.63 – 0.94)	1.02 (0.84 – 1.05)	NS
$ADCsk^{0}_{20\%}$	1.01 (0.93 – 1.1)	1.27 (1.13 – 1.28)	NS
$ADCsk^{0}_{40\%}$	1.26 (1.06 – 1.59)	1.48 (1.32 – 1.51)	NS
$ADCsk^{0}_{60\%}$	1.49 (1.38 – 1.77)	1.75 (1.41 – 1.8)	NS
$ADCsk^{0}_{80\%}$	1.46 (1.29 – 1.77)	1.55 (1.53 – 1.57)	NS
ADCkurt [×10⁻⁶ mm²/s]			
$ADCkurt^{0}_{5\%}$	1.6 (1.21 – 1.76)	2.19 (1.83 – 2.21)	NS
$ADCkurt^{0}_{10\%}$	1.81 (1.44 – 2.09)	2.5 (2.25 – 2.68)	NS
$ADCkurt^{0}_{20\%}$	1.02 (0.62 – 1.2)	1.47 (1.37 – 1.47)	NS
$ADCkurt^{0}_{40\%}$	1.01 (0.43 – 2.49)	1.59 (0.89 – 2.34)	NS
$ADCkurt^{0}_{60\%}$	1.78 (1.37 – 3.31)	2.88 (1.04 – 3.82)	NS
$ADCkurt^{0}_{80\%}$	2.08 (1.45 – 3.62)	2.6 (1.64 – 3.01)	NS
ADCentr [×10⁻⁶ mm²/s]			
$ADCentr^{0}_{5\%}$	8.19 (8.16 – 8.29)	8.26 (8.2 – 8.3)	NS
$ADCentr^{0}_{10\%}$	8.35 (8.27 – 8.38)	8.33 (8.29 – 8.36)	NS
$ADCentr^{0}_{20\%}$	8.41 (8.35 – 8.52)	8.48 (8.37 – 8.49)	NS
$ADCentr^{0}_{40\%}$	8.22 (8.13 – 8.32)	8.34 (8.16 – 8.38)	NS
$ADCentr^{0}_{60\%}$	8.05 (7.99 – 8.14)	8.12 (7.97 – 8.19)	NS
$ADCentr^{0}_{80\%}$	8.01 (7.87 – 8.11)	8.03 (7.9 – 8.06)	NS
**T1 PARAMETERS TO PREDICT T2 RESPONSE**			
DV [cm³]			
$DV^{1}_{5\%}$	37169.7 (32037.88 – 39925.08)	28921.4 (25664.82 – 37200.73)	NS
$DV^{1}_{10\%}$	17184.2 (15067.2 – 19655.07)	14336.8 (14287.98 – 16419.55)	NS
$DV^{1}_{20\%}$	4481 (3756.58 – 5136.95)	4690.6 (4484.5 – 5029.38)	NS
$DV^{1}_{40\%}$	1942.5 (1849.07 – 2129.77)	2235.5 (2169.05 – 2393.9)	NS
$DV^{1}_{60\%}$	1479 (1384.98 – 1570.05)	1568.5 (1434.78 – 1590.4)	NS
$DV^{1}_{80\%}$	1123.54 (1057.52 – 1181.45)	1145.1 (1080.23 – 1190.1)	NS
ADCmean [×10⁻⁶ mm²/s]			
$ADCmean^{1}_{5\%}$	1084.82 (1013.89 – 1109.41)	1153.58 (1056.11 – 1204.95)	NS
$ADCmean^{1}_{10\%}$	1177.98 (1062.83 – 1203.3)	1211.03 (1117.46 – 1268.56)	NS
$ADCmean^{1}_{20\%}$	1309.41 (1245.77 – 1356.17)	1208.16 (1135.33 – 1255.09)	NS
$ADCmean^{1}_{40\%}$	1239.24 (1153.79 – 1299.64)	1201.56 (1111.8 – 1208.2)	NS

$ADCmean^{1}_{60\%}$	1144.3 (1068.9 – 1209.41)	1092.96 (1065.8 – 1129.68)	NS
$ADCmean^{1}_{80\%}$	1099.22 (1038.19 – 1184.59)	1085.08 (1064.47 – 1089.78)	NS
***ADCsd* [×10⁻⁶ mm²/s]**			
$ADCsd^{1}_{5\%}$	575.05 (558.95 – 604.19)	555.59 (530.27 – 556.02)	NS
$ADCsd^{1}_{10\%}$	615.13 (578.01 – 639.14)	551.7 (536.78 – 595.08)	NS
$ADCsd^{1}_{20\%}$	690.32 (633 – 735.15)	633.84 (598.02 – 646.79)	NS
$ADCsd^{1}_{40\%}$	649.79 (604.49 – 679.71)	573.36 (526.2 – 611.74)	NS
$ADCsd^{1}_{60\%}$	554.06 (492.59 – 600.81)	503.01 (464.44 – 538.4)	NS
$ADCsd^{1}_{80\%}$	488.4 (452.14 – 543.79)	472.01 (429.54 – 489.52)	NS
***ADCmd* [×10^{-6} mm^2/s]**			
$ADCmd^{1}_{5\%}$	1041.75 (931.5 – 1120.5)	1174.5 (1053 – 1235.25)	NS
$ADCmd^{1}_{10\%}$	1174.5 (1032.75 – 1228.5)	1228.5 (1086.75 – 1289.25)	NS
$ADCmd^{1}_{20\%}$	1174.5 (1113.75 – 1228.5)	1093.5 (972 – 1154.25)	NS
$ADCmd^{1}_{40\%}$	985.5 (951.75 – 1073.25)	985.5 (938.63 – 1005.75)	NS
$ADCmd^{1}_{60\%}$	923 (887.5 – 975)	924 (915.38 – 929.63)	NS
$ADCmd^{1}_{80\%}$	924 (906.38 – 982.13)	924 (915.38 – 924)	NS
***ADC5p* [×10^{-6} mm^2/s]**			
$ADC5p^{1}_{5\%}$	256.5 (236.25 – 310.5)	337.5 (236.25 – 357.75)	NS
$ADC5p^{1}_{10\%}$	283.5 (249.75 – 330.75)	445.5 (283.5 – 445.5)	NS
$ADC5p^{1}_{20\%}$	445.5 (391.5 – 492.75)	445.5 (364.5 – 506.25)	NS
$ADC5p^{1}_{40\%}$	607.5 (499.5 – 634.5)	553.5 (518.63 – 594)	NS
$ADC5p^{1}_{60\%}$	612.5 (611 – 635.25)	612 (608.63 – 612.38)	NS
$ADC5p^{1}_{80\%}$	625.25 (612 – 637.13)	612.5 (612.13 – 648.13)	NS
***ADC95p* [×10^{-6} mm^2/s]**			
$ADC95p^{1}_{5\%}$	1957.5 (1950.75 – 2072.25)	1930.5 (1930.5 – 2052)	NS
$ADC95p^{1}_{10\%}$	2225.25 (2119.5 – 2261.25)	2146.5 (2085.75 – 2146.5)	NS
$ADC95p^{1}_{20\%}$	2686.5 (2416.5 – 2902.5)	2335.5 (2254.5 – 2376)	0.032
$ADC95p^{1}_{40\%}$	2632.5 (2369.25 – 2743.88)	2254.5 (2133.38 – 2315.25)	NS
$ADC95p^{1}_{60\%}$	2389.5 (2124.75 – 2475)	2196 (2058.38 – 2239.88)	NS
$ADC95p^{1}_{80\%}$	2149.42 (2027.88 – 2334)	2124 (1998 – 2152.88)	NS
***ADCsk* [×10^{-6} mm^2/s]**			
$ADCsk^{1}_{5\%}$	0.84 (0.72 – 0.88)	0.6 (0.53 – 0.74)	NS
$ADCsk^{1}_{10\%}$	0.9 (0.71 – 1.04)	0.82 (0.69 – 0.9)	NS
$ADCsk^{1}_{20\%}$	1.02 (0.93 – 1.1)	1.08 (0.86 – 1.18)	NS
$ADCsk^{1}_{40\%}$	1.29 (1.05 – 1.56)	1.3 (1.08 – 1.42)	NS
$ADCsk^{1}_{60\%}$	1.56 (1.36 – 1.81)	1.48 (1.47 – 1.7)	NS
$ADCsk^{1}_{80\%}$	1.47 (1.33 – 1.65)	1.48 (1.4 – 1.67)	NS
***ADCkurt* [×10^{-6} mm^2/s]**			
$ADCkurt^{1}_{5\%}$	1.82 (1.23 – 2.07)	1.62 (1.28 – 1.74)	NS
$ADCkurt^{1}_{10\%}$	2.03 (1.29 – 2.14)	1.84 (1.76 – 2.19)	NS
$ADCkurt^{1}_{20\%}$	0.94 (0.58 – 1.46)	1.52 (0.76 – 1.54)	NS
$ADCkurt^{1}_{40\%}$	1.42 (0.6 – 1.96)	1.74 (0.81 – 2.21)	NS

$ADCkurt^{1}_{60\%}$	2.47 (1.37 – 3.41)	2.18 (2.03 – 3.28)	NS
$ADCkurt^{1}_{80\%}$	2.28 (1.35 – 3.23)	2.15 (1.84 – 3.24)	NS
***ADCentr* [×10⁻⁶ mm²/s]**			
$ADCentr^{1}_{5\%}$	8.24 (8.19 – 8.29)	8.2 (8.19 – 8.27)	NS
$ADCentr^{1}_{10\%}$	8.36 (8.31 – 8.38)	8.32 (8.28 – 8.35)	NS
$ADCentr^{1}_{20\%}$	8.48 (8.41 – 8.54)	8.41 (8.32 – 8.46)	NS
$ADCentr^{1}_{40\%}$	8.26 (8.16 – 8.36)	8.21 (8.14 – 8.27)	NS
$ADCentr^{1}_{60\%}$	8.06 (7.94 – 8.17)	8.03 (7.94 – 8.08)	NS
$ADCentr^{1}_{80\%}$	8 (7.86 – 8.15)	8.01 (7.89 – 8.01)	NS

Table S3. Median values, Interquartile ranges (IQR) and results of Mann-Whitney U test of parameters for prediction of final response to treatment (evaluating percentage differences between T1 and T0 parameters and using Lugano on T2 images).

T1-T0 PARAMETERS TO PREDICT T2 RESPONSE			
**Parameters**	**Median (IQR) - CMR**	**Median (IQR) - PMR**	**p**
***ΔDV* [%]**			
$\Delta DV^{01}_{5\%}$	1.21 (-6.7 – 6.78)	-1.2 (-7.73 – 11.75)	NS
$\Delta DV^{01}_{10\%}$	8.83 (1.2 – 17.81)	4.28 (1.08 – 6.18)	NS
$\Delta DV^{01}_{20\%}$	10.33 (-0.48 – 17.67)	2.4 (-0.83 – 16.61)	NS
$\Delta DV^{01}_{40\%}$	-1.05 (-7.64 – 3.56)	-12.23 (-12.94 – -2.78)	NS
$\Delta DV^{01}_{60\%}$	-1.57 (-5.53 – 2.03)	-10.65 (-15.13 – -3.35)	NS
$\Delta DV^{01}_{80\%}$	-0.94 (-5.31 – 5.97)	-6.78 (-10.36 – -3.38)	NS
**$\Delta ADCmean$ [%]**	-3.64 (-5.72 – 0.09)	-3.81 (-6.67 – -1.08)	
$\Delta ADCmean^{01}_{5\%}$	-4.46 (-6.65 – -0.27)	-2 (-6.02 – -1.33)	NS
$\Delta ADCmean^{01}_{10\%}$	-4.31 (-10.21 – -0.46)	-11.11 (-12.55 – -6.21)	NS
$\Delta ADCmean^{01}_{20\%}$	0.92 (-10.99 – 7.82)	-5.8 (-8.03 – -4.6)	NS
$\Delta ADCmean^{01}_{40\%}$	-0.05 (-4.54 – 3.75)	-3.27 (-9.93 – 0.66)	NS
$\Delta ADCmean^{01}_{60\%}$	1.35 (-3.01 – 3.75)	-0.55 (-7.11 – 2.64)	NS
$\Delta ADCmean^{01}_{80\%}$			NS
**$\Delta ADCsd$ [%]**	4.18 (0.76 – 5.22)	-3.66 (-5.46 – -0.67)	
$\Delta ADCsd^{01}_{5\%}$	1.44 (-2.52 – 5.08)	-0.23 (-6.75 – 0.36)	NS
$\Delta ADCsd^{01}_{10\%}$	7.02 (-6 – 12.41)	-10.37 (-13.97 – -1.59)	NS
$\Delta ADCsd^{01}_{20\%}$	7.49 (-3.5 – 11.39)	-21.52 (-22.4 – -7.57)	NS
$\Delta ADCsd^{01}_{40\%}$	3.17 (-4.49 – 8.95)	-16.29 (-26.24 – -3.56)	NS
$\Delta ADCsd^{01}_{60\%}$	1.73 (-0.98 – 3.5)	-1.91 (-20.36 – 3.72)	NS
$\Delta ADCsd^{01}_{80\%}$			NS
**$\Delta ADCmd$ [%]**	-5.48 (-9.52 – -2.17)	-4.4 (-6.65 – 0.55)	
$\Delta ADCmd^{01}_{5\%}$	-2.6 (-8.67 – 0.52)	-2.15 (-9.16 – -0.54)	NS
$\Delta ADCmd^{01}_{10\%}$	-2.3 (-9.62 – 2.27)	-10.99 (-15.4 – -0.98)	NS
$\Delta ADCmd^{01}_{20\%}$	-3.53 (-11.16 – 4.07)	0 (-2.05 – 0)	NS
$\Delta ADCmd^{01}_{40\%}$	-0.73 (-3.29 – 3.7)	1.26 (-1.07 – 2.43)	NS
$\Delta ADCmd^{01}_{60\%}$	-0.48 (-4.17 – 2.51)	0 (-0.93 – 1.28)	NS
$\Delta ADCmd^{01}_{80\%}$			NS

**$\Delta ADC5p$ [%]**	-2.21 (-15.89 – 2)	-13.79 (-19.24 – 2.55)	
$\Delta ADC5p_{5\%}^{01}$	-5.63 (-17.56 – 7.02)	0 (-7.89 – 4.84)	NS
$\Delta ADC5p_{10\%}^{01}$	-23.6 (-33.6 – -11.62)	-19.51 (-38.21 – -4.88)	NS
$\Delta ADC5p_{20\%}^{01}$	-7.8 (-15.12 – 0)	4.65 (-8.84 – 5.01)	NS
$\Delta ADC5p_{40\%}^{01}$	0.25 (-0.47 – 4.23)	4.17 (0.61 – 4.23)	NS
$\Delta ADC5p_{60\%}^{01}$	0.69 (0.36 – 3.94)	4.08 (-1.75 – 10.42)	NS
$\Delta ADC5p_{80\%}^{01}$			NS
**$\Delta ADC95p$ [%]**	-1.36 (-2.57 – 3.23)	-2.72 (-3.7 – -1.64)	
$\Delta ADC95p_{5\%}^{01}$	0 (-6.22 – 3.01)	-2.55 (-6.71 – -2.48)	NS
$\Delta ADC95p_{10\%}^{01}$	0.94 (-7.98 – 5.86)	-19.91 (-22.31 – -6.75)	NS
$\Delta ADC95p_{20\%}^{01}$	2.79 (-7.7 – 11.6)	-23.79 (-25.77 – -7.77)	NS
$\Delta ADC95p_{40\%}^{01}$	1.36 (-5.38 – 6.47)	-12.6 (-21.84 – -2.21)	NS
$\Delta ADC95p_{60\%}^{01}$	0.96 (-0.58 – 3.53)	1.14 (-18.63 – 4.52)	NS
$\Delta ADC95p_{80\%}^{01}$			NS
**$\Delta ADCsk$ [%]**	19.44 (-15.33 – 31.52)	-11.28 (-25.57 – -10.53)	
$\Delta ADCsk_{5\%}^{01}$	24.25 (-21.31 – 41.93)	-12.61 (-30.46 – 0.91)	NS
$\Delta ADCsk_{10\%}^{01}$	0.8 (-9.19 – 9.13)	-15.44 (-32.2 – 5.69)	NS
$\Delta ADCsk_{20\%}^{01}$	1.77 (-7.54 – 21.65)	-3.7 (-24.52 – 1.1)	NS
$\Delta ADCsk_{40\%}^{01}$	4.97 (-2.51 – 16.44)	-2.8 (-12.74 – 9.92)	NS
$\Delta ADCsk_{60\%}^{01}$	0.55 (-7.66 – 15.25)	-3.01 (-9.45 – 6.51)	NS
$\Delta ADCsk_{80\%}^{01}$			NS
**$\Delta ADCkurt$ [%]**	20.32 (-39.07 – 47.14)	-25.97 (-29.97 – -21.54)	
$\Delta ADCkurt_{5\%}^{01}$	-1.5 (-29.52 – 32)	-26.17 (-33.99 – -1.87)	NS
$\Delta ADCkurt_{10\%}^{01}$	-24.38 (-38.94 – -2.55)	5.1 (-47.6 – 11.31)	NS
$\Delta ADCkurt_{20\%}^{01}$	-33.5 (-91.6 – 3.12)	-8.66 (-53.51 – 120.75)	NS
$\Delta ADCkurt_{40\%}^{01}$	-3.94 (-36.17 – 35.02)	-11.65 (-26.22 – 309.74)	NS
$\Delta ADCkurt_{60\%}^{01}$	0.89 (-24.02 – 32.37)	14.59 (-21.15 – 50.91)	NS
$\Delta ADCkurt_{80\%}^{01}$			NS
**$\Delta ADCentr$ [%]**	0.31 (-0.12 – 0.49)	-0.29 (-0.61 – 0.01)	
$\Delta ADCentr_{5\%}^{01}$	0.18 (0.01 – 0.53)	-0.11 (-0.41 – 0.13)	NS
$\Delta ADCentr_{10\%}^{01}$	0.7 (-0.63 – 1.1)	-0.48 (-0.74 – -0.22)	NS
$\Delta ADCentr_{20\%}^{01}$	0.82 (-0.73 – 1.29)	-1.29 (-1.52 – -0.2)	NS
$\Delta ADCentr_{40\%}^{01}$	0 (-1.11 – 1)	-1.06 (-1.32 – -0.33)	NS
$\Delta ADCentr_{60\%}^{01}$	0.23 (-0.85 – 0.56)	-0.15 (-0.62 – -0.08)	NS
$\Delta ADCentr_{80\%}^{01}$	1.21 (-6.7 – 6.78)	-1.2 (-7.73 – 11.75)	NS

Assessment of intermediate response to treatment

Table S4. Median values, Interquartile ranges (IQR) and results of Mann-Whitney U test of parameters for assessment of intermediate response to treatment (evaluating T1 parameters and using Lugano on T1 images).

T1 PARAMETERS TO ASSESS T1 RESPONSE			
**Parameters**	**Median (IQR) - CMR**	**Median (IQR) - PMR**	**p**

DV [cm³]			
$DV^1_{5\%}$	36563.25 (31272.35 – 39038.7)	27766.55 (26278.5 – 39927.4)	NS
$DV^1_{10\%}$	16613.56 (14683.8 – 18551.35)	15644.1 (14336.8 – 17113.8)	NS
$DV^1_{20\%}$	4328.54 (3727.75 – 4721)	4932.35 (4690.6 – 5258.5)	NS
$DV^1_{40\%}$	1916.35 (1846.85 – 2084.55)	2311.45 (2235.5 – 2446.7)	0.005
$DV^1_{60\%}$	1492.25 (1377.65 – 1572.9)	1534.45 (1404.5 – 1597.7)	NS
$DV^1_{80\%}$	1129.48 (1078.5 – 1206.1)	1138.15 (1058.6 – 1205.1)	NS
ADCmean [×10⁻⁶ mm²/s]			
$ADCmean^1_{5\%}$	1086.39 (1002.85 – 1110.33)	1066.63 (1023.62 – 1153.58)	NS
$ADCmean^1_{10\%}$	1171.83 (1059.73 – 1205.33)	1156.96 (1086.27 – 1211.03)	NS
$ADCmean^1_{20\%}$	1311.58 (1252.74 – 1356.32)	1221.34 (1111.06 – 1240.01)	0.009
$ADCmean^1_{40\%}$	1234.67 (1152.78 – 1295)	1168.54 (1081.88 – 1210.41)	NS
$ADCmean^1_{60\%}$	1118.52 (1065.35 – 1191.82)	1074.86 (1030.19 – 1141.92)	NS
$ADCmean^1_{80\%}$	1089.77 (1035.38 – 1158.51)	1071.34 (981.93 – 1091.35)	NS
ADCsd [×10⁻⁶ mm²/s]			
$ADCsd^1_{5\%}$	575.36 (556.25 – 605.6)	559.15 (555.59 – 572.69)	NS
$ADCsd^1_{10\%}$	620.58 (576.4 – 639.3)	575.04 (551.7 – 587.23)	NS
$ADCsd^1_{20\%}$	695.49 (644.57 – 737.93)	609.96 (577.22 – 635.21)	0.009
$ADCsd^1_{40\%}$	646 (602.52 – 676.68)	572.03 (521.82 – 624.54)	NS
$ADCsd^1_{60\%}$	553.91 (481.17 – 587.12)	506.22 (452.49 – 550.19)	NS
$ADCsd^1_{80\%}$	482.96 (432.5 – 532.92)	475.81 (415.38 – 495.36)	NS
ADCmd [×10⁻⁶ mm²/s]			
$ADCmd^1_{5\%}$	1067.73 (931.5 – 1120.5)	1039.5 (1012.5 – 1174.5)	NS
$ADCmd^1_{10\%}$	1152.41 (1026 – 1228.5)	1161 (1039.5 – 1228.5)	NS
$ADCmd^1_{20\%}$	1170.82 (1107 – 1228.5)	1093.5 (1012.5 – 1174.5)	NS
$ADCmd^1_{40\%}$	985.5 (945 – 1033.75)	958.5 (923 – 1012.5)	NS
$ADCmd^1_{60\%}$	913.75 (887.5 – 950)	918.25 (871 – 931.5)	NS
$ADCmd^1_{80\%}$	918.5 (904.25 – 967.75)	918.25 (852 – 924)	NS
ADC5p [×10⁻⁶ mm²/s]			
$ADC5p^1_{5\%}$	256.5 (216 – 310.5)	229.5 (202.5 – 337.5)	NS
$ADC5p^1_{10\%}$	283.5 (243 – 337.5)	270 (229.5 – 445.5)	NS
$ADC5p^1_{20\%}$	445.5 (391.5 – 513)	432 (391.5 – 526.5)	NS
$ADC5p^1_{40\%}$	607.5 (537.75 – 634.5)	530.25 (472.5 – 607.5)	NS
$ADC5p^1_{60\%}$	618.8 (611 – 636)	609.75 (562.5 – 612.5)	NS
$ADC5p^1_{80\%}$	630.64 (612 – 638.25)	612.25 (586.5 – 660)	NS
ADC95p [×10⁻⁶ mm²/s]			
$ADC95p^1_{5\%}$	1957.5 (1944 – 2079)	1930.5 (1930.5 – 2038.5)	NS
$ADC95p^1_{10\%}$	2243.45 (2119.5 – 2268)	2106 (2038.5 – 2146.5)	NS
$ADC95p^1_{20\%}$	2686.5 (2497.5 – 2902.5)	2281.5 (2227.5 – 2389.5)	0.001
$ADC95p^1_{40\%}$	2646 (2349 – 2796.75)	2214 (2093 – 2335.5)	0.013

$ADC95p^{1}_{60\%}$	2388.5 (2087 – 2462.5)	2105.5 (2012.5 – 2254.5)	NS
$ADC95p^{1}_{80\%}$	2137.16 (1976.75 – 2302.25)	2040 (1956 – 2162.5)	NS
ADCsk [×10⁻⁶ mm²/s]			
$ADCsk^{1}_{5\%}$	0.85 (0.74 – 0.89)	0.74 (0.6 – 0.82)	NS
$ADCsk^{1}_{10\%}$	0.91 (0.79 – 1.04)	0.8 (0.65 – 0.85)	NS
$ADCsk^{1}_{20\%}$	1.02 (0.94 – 1.12)	1.11 (0.79 – 1.21)	NS
$ADCsk^{1}_{40\%}$	1.33 (1.13 – 1.56)	1.38 (1.01 – 1.48)	NS
$ADCsk^{1}_{60\%}$	1.64 (1.42 – 1.82)	1.62 (1.47 – 1.83)	NS
$ADCsk^{1}_{80\%}$	1.52 (1.35 – 1.71)	1.59 (1.37 – 1.73)	NS
ADCkurt [×10⁻⁶ mm²/s]			
$ADCkurt^{1}_{5\%}$	1.87 (1.29 – 2.07)	1.7 (1.17 – 2.01)	NS
$ADCkurt^{1}_{10\%}$	2.04 (1.6 – 2.19)	1.91 (1.74 – 2.3)	NS
$ADCkurt^{1}_{20\%}$	0.98 (0.63 – 1.49)	1.53 (0.51 – 1.87)	NS
$ADCkurt^{1}_{40\%}$	1.47 (0.71 – 2.18)	2.02 (0.5 – 2.36)	NS
$ADCkurt^{1}_{60\%}$	2.61 (1.79 – 3.59)	2.92 (1.99 – 3.97)	NS
$ADCkurt^{1}_{80\%}$	2.46 (1.64 – 3.29)	2.82 (1.74 – 3.61)	NS
ADCentr [×10⁻⁶ mm²/s]			
$ADCentr^{1}_{5\%}$	8.22 (8.19 – 8.27)	8.25 (8.2 – 8.29)	NS
$ADCentr^{1}_{10\%}$	8.35 (8.3 – 8.39)	8.33 (8.29 – 8.35)	NS
$ADCentr^{1}_{20\%}$	8.49 (8.4 – 8.54)	8.37 (8.3 – 8.47)	0.024
$ADCentr^{1}_{40\%}$	8.26 (8.15 – 8.33)	8.18 (8.12 – 8.29)	NS
$ADCentr^{1}_{60\%}$	8.02 (7.92 – 8.15)	8.02 (7.91 – 8.1)	NS
$ADCentr^{1}_{80\%}$	7.99 (7.85 – 8.1)	8 (7.85 – 8.01)	NS

Table S5. Median values, Interquartile ranges (IQR) and results of Mann-Whitney U test of parameters for assessment of intermediate response to treatment (evaluating percentage differences between T1 and T0 parameters and using Lugano on T1 images).

T1-T0 PARAMETERS TO ASSESS T1 RESPONSE			
**Parameters**	**Median (IQR) - CMR**	**Median (IQR) - PMR**	**p**
ΔDV [%]			
$\Delta DV^{01}_{5\%}$	0.71 (-7.15 – 7.4)	3.69 (-3.38 – 8.39)	NS
$\Delta DV^{01}_{10\%}$	7.35 (1.07 – 20.28)	5.54 (3.22 – 8.21)	NS
$\Delta DV^{01}_{20\%}$	7.91 (-3.28 – 18.06)	17.04 (1.32 – 21.01)	NS
$\Delta DV^{01}_{40\%}$	-3.51 (-8.16 – 1.12)	0.37 (-12.47 – 17.37)	NS
$\Delta DV^{01}_{60\%}$	-1.77 (-5.59 – 1.01)	-0.92 (-12.14 – 2.73)	NS
$\Delta DV^{01}_{80\%}$	-2.13 (-5.43 – 4.76)	-3.29 (-7.98 – -0.3)	NS
ΔADCmean [%]			
$\Delta ADCmean^{01}_{5\%}$	-2.4 (-5.28 – 0.21)	-3.81 (-11.61 – -2.34)	NS
$\Delta ADCmean^{01}_{10\%}$	-2.51 (-5.97 – 0.58)	-2 (-11.78 – -0.77)	NS
$\Delta ADCmean^{01}_{20\%}$	-4.12 (-7.93 – -0.22)	-11.11 (-16.21 – -4.5)	NS
$\Delta ADCmean^{01}_{40\%}$	1.29 (-8.85 – 8.14)	-5.8 (-9.96 – -4.09)	NS
$\Delta ADCmean^{01}_{60\%}$	0.27 (-4.93 – 4.47)	-3.27 (-5.62 – -0.87)	NS

$\Delta ADCmean_{80\%}^{01}$	1.85 (-1.34 – 3.81)	-0.83 (-5.27 – 0.51)	NS
**$\Delta ADCsd$ [%]**			
$\Delta ADCsd_{5\%}^{01}$	4.85 (1.87 – 5.66)	-4.79 (-9.43 – -2.67)	0.006
$\Delta ADCsd_{10\%}^{01}$	2.69 (-0.08 – 5.12)	-7.38 (-10.08 – -0.04)	0.036
$\Delta ADCsd_{20\%}^{01}$	8.47 (-2.99 – 12.54)	-10.37 (-14.92 – -5.93)	0.013
$\Delta ADCsd_{40\%}^{01}$	7.76 (-0.09 – 12)	-9.52 (-21.81 – -2.84)	0.019
$\Delta ADCsd_{60\%}^{01}$	3.18 (-4.57 – 9.62)	-3.02 (-19.61 – 0.83)	NS
$\Delta ADCsd_{80\%}^{01}$	1.73 (-0.52 – 4.56)	-1.91 (-8.34 – 2.17)	NS
**$\Delta ADCmd$ [%]**			
$\Delta ADCmd_{5\%}^{01}$	-4.65 (-8.15 – -2.15)	-4.94 (-11.87 – -2.75)	NS
$\Delta ADCmd_{10\%}^{01}$	-2.4 (-6.32 – 1.03)	-2.3 (-14.71 – -1.61)	NS
$\Delta ADCmd_{20\%}^{01}$	-0.05 (-8.02 – 3.08)	-10.99 (-19.68 – -2.94)	NS
$\Delta ADCmd_{40\%}^{01}$	-1.09 (-8.07 – 5.25)	-2.74 (-8.66 – 0)	NS
$\Delta ADCmd_{60\%}^{01}$	-0.11 (-2.18 – 3.75)	-1.84 (-4.19 – 1.65)	NS
$\Delta ADCmd_{80\%}^{01}$	-0.02 (-3.98 – 3.07)	-1.24 (-4.01 – 0.43)	NS
**$\Delta ADC5p$ [%]**			
$\Delta ADC5p_{5\%}^{01}$	0 (-11.69 – 4)	-13.79 (-21.79 – 2)	NS
$\Delta ADC5p_{10\%}^{01}$	-0.72 (-15.69 – 7.15)	0 (-17.35 – 8.17)	NS
$\Delta ADC5p_{20\%}^{01}$	-22.61 (-29.61 – -10.46)	-19.51 (-41.72 – 7.69)	NS
$\Delta ADC5p_{40\%}^{01}$	-3.88 (-11.06 – 0)	4.08 (-14.65 – 4.77)	NS
$\Delta ADC5p_{60\%}^{01}$	0.08 (-0.47 – 3.92)	4.17 (-0.14 – 4.58)	NS
$\Delta ADC5p_{80\%}^{01}$	0.58 (0.32 – 2.4)	3.99 (-0.92 – 6.19)	NS
**$\Delta ADC95p$ [%]**			
$\Delta ADC95p_{5\%}^{01}$	0.44 (-1.41 – 3.49)	-2.72 (-8.86 – -2.36)	0.045
$\Delta ADC95p_{10\%}^{01}$	0 (-3.73 – 3.33)	-2.58 (-11.68 – -2.52)	NS
$\Delta ADC95p_{20\%}^{01}$	2.8 (-5.56 – 6.43)	-18.43 (-20.71 – -6.46)	0.013
$\Delta ADC95p_{40\%}^{01}$	4.65 (-3.64 – 13.14)	-10.33 (-24.45 – -1.82)	0.02
$\Delta ADC95p_{60\%}^{01}$	1.95 (-4.34 – 6.81)	-3.85 (-15.68 – 0.31)	0.048
$\Delta ADC95p_{80\%}^{01}$	1.47 (-0.11 – 3.95)	-1.33 (-9.29 – 2.27)	NS
**$\Delta ADCsk$ [%]**			
$\Delta ADCsk_{5\%}^{01}$	14.81 (-16.46 – 32.27)	-10.29 (-16.04 – 19.83)	NS
$\Delta ADCsk_{10\%}^{01}$	17.49 (-21.6 – 37.74)	5.41 (-18.56 – 16.53)	NS
$\Delta ADCsk_{20\%}^{01}$	-0.5 (-9.24 – 8.36)	12.73 (-21.03 – 58.18)	NS
$\Delta ADCsk_{40\%}^{01}$	-0.33 (-9.3 – 6.89)	2.69 (-10.64 – 44.6)	NS
$\Delta ADCsk_{60\%}^{01}$	4.52 (-3.19 – 10.31)	14.16 (-6.11 – 30.01)	NS
$\Delta ADCsk_{80\%}^{01}$	0.33 (-8.08 – 6.89)	9.69 (-5.16 – 23.52)	NS
**$\Delta ADCkurt$ [%]**			
$\Delta ADCkurt_{5\%}^{01}$	10.08 (-39.79 – 26.63)	-20.06 (-27.3 – 83.88)	NS
$\Delta ADCkurt_{10\%}^{01}$	-2.62 (-31.2 – 24.52)	6.23 (-28.78 – 56.62)	NS
$\Delta ADCkurt_{20\%}^{01}$	-19.02 (-36.71 – -1.09)	5.1 (-146.41 – 40.59)	NS
$\Delta ADCkurt_{40\%}^{01}$	-33.22 (-64.47 – 1.69)	-8.66 (-80.48 – 124.27)	NS
$\Delta ADCkurt_{60\%}^{01}$	2.15 (-15.79 – 36.29)	-11.65 (-61.64 – 165.75)	NS
$\Delta ADCkurt_{80\%}^{01}$	0.93 (-19.2 – 35.46)	14.59 (-48.35 – 48.78)	NS

$\Delta ADCentr$ [%]			
$\Delta ADCentr^{01}_{5\%}$	0.32 (-0.04 – 0.56)	-0.53 (-1.24 – -0.19)	0.014
$\Delta ADCentr^{01}_{10\%}$	0.27 (0.16 – 0.56)	-0.51 (-1.16 – -0.03)	0.02
$\Delta ADCentr^{01}_{20\%}$	0.71 (-0.27 – 1.21)	-0.83 (-2.16 – -0.39)	0.009
$\Delta ADCentr^{01}_{40\%}$	0.88 (-0.18 – 1.36)	-1.42 (-1.65 – -0.92)	0.014
$\Delta ADCentr^{01}_{60\%}$	0.17 (-1.23 – 1.04)	-1.06 (-1.33 – -0.69)	NS
$\Delta ADCentr^{01}_{80\%}$	0.28 (-0.57 – 0.6)	-0.77 (-0.79 – -0.12)	NS

Assessment of final response to treatment

Table S6. Median values, Interquartile ranges (IQR) and results of Mann-Whitney U test of parameters for assessment of final response to treatment (evaluating T2 parameters and using Lugano on T2 images).

T2 PARAMETERS TO ASSESS T2 RESPONSE			
**Parameters**	**Median (IQR) - CMR**	**Median (IQR) - PMR**	**p**
DV [cm³]			
$DV^{2}_{5\%}$	36639.3 (32221.55 – 39613.35)	29806.2 (28040.48 – 39216.08)	NS
$DV^{2}_{10\%}$	19067.73 (15787.88 – 20550.9)	13742.1 (13155.9 – 20846.03)	NS
$DV^{2}_{20\%}$	5349.65 (4187.88 – 6640.92)	4964.4 (4231.28 – 4971.75)	NS
$DV^{2}_{40\%}$	2314.3 (1978.8 – 2968.17)	2200.2 (1960.87 – 2398.35)	NS
$DV^{2}_{60\%}$	1609.2 (1425.65 – 1793.85)	1596.7 (1393.75 – 1677.18)	NS
$DV^{2}_{80\%}$	1168.55 (1042.85 – 1277.55)	1173 (1030.95 – 1198.88)	NS
ADCmean [×10⁻⁶ mm²/s]			
$ADCmean^{2}_{5\%}$	1103.54 (1061.37 – 1159.35)	1211.08 (1180.53 – 1227.21)	0.014
$ADCmean^{2}_{10\%}$	1193.06 (1133.5 – 1269.54)	1287.27 (1232.2 – 1289.43)	NS
$ADCmean^{2}_{20\%}$	1282.06 (1189.5 – 1350.87)	1343.33 (1113.85 – 1365.01)	NS
$ADCmean^{2}_{40\%}$	1177.46 (1101.63 – 1223.81)	1208.4 (1075.42 – 1294.31)	NS
$ADCmean^{2}_{60\%}$	1099.87 (1034.36 – 1140.35)	1044.34 (1035.87 – 1135.78)	NS
$ADCmean^{2}_{80\%}$	1097.1 (1023.92 – 1144.04)	1048.23 (349.9 – 1058.31)	NS
ADCsd [×10⁻⁶ mm²/s]			
$ADCsd^{2}_{5\%}$	560.03 (545.76 – 593.15)	591.45 (557.52 – 611.81)	NS
$ADCsd^{2}_{10\%}$	592.19 (556.17 – 622.25)	573.08 (556.69 – 643.29)	NS
$ADCsd^{2}_{20\%}$	653.58 (618.96 – 700.45)	602.71 (581.09 – 752.7)	NS
$ADCsd^{2}_{40\%}$	643.57 (583.64 – 667.85)	534.2 (493.48 – 678.09)	NS
$ADCsd^{2}_{60\%}$	545.4 (489.41 – 587.97)	421.81 (421.34 – 555.8)	NS
$ADCsd^{2}_{80\%}$	489.19 (410.92 – 551.47)	452.85 (416.78 – 501.36)	NS
ADCmd [×10⁻⁶ mm²/s]			
$ADCmd^{2}_{5\%}$	1066.5 (1034.44 – 1174.5)	1228.5 (1208.25 – 1289.25)	0.01
$ADCmd^{2}_{10\%}$	1174.5 (1093.5 – 1282.5)	1282.5 (1262.25 – 1363.5)	NS
$ADCmd^{2}_{20\%}$	1149.75 (1059.75 – 1201.5)	1174.5 (951.75 – 1275.75)	NS
$ADCmd^{2}_{40\%}$	931.5 (891 – 965.25)	1037.5 (917.5 – 1059.25)	NS
$ADCmd^{2}_{60\%}$	877.5 (864 – 906.5)	900 (890.63 – 936.75)	NS
$ADCmd^{2}_{80\%}$	924 (871.13 – 947.63)	924 (924 – 929.63)	NS

ADC5p [×10⁻⁶ mm²/s]			
$ADC5p^2_{5\%}$	310.5 (256.5 – 337.5)	337.5 (317.25 – 337.5)	NS
$ADC5p^2_{10\%}$	310.5 (310.5 – 364.5)	337.5 (317.25 – 378)	NS
$ADC5p^2_{20\%}$	553.5 (411.75 – 587.25)	472.5 (411.75 – 492.75)	NS
$ADC5p^2_{40\%}$	580.5 (533.25 – 614.25)	607.5 (506.25 – 648.75)	NS
$ADC5p^2_{60\%}$	607.5 (560.25 – 612.5)	636 (618.38 – 636.75)	NS
$ADC5p^2_{80\%}$	612 (610.88 – 633)	612 (610.13 – 648)	NS
ADC95p [$\times 10^{-6}$ mm^2/s]			
$ADC95p^2_{5\%}$	1984.5 (1890.86 – 2072.25)	2065.5 (1984.5 – 2106)	NS
$ADC95p^2_{10\%}$	2173.5 (2004.75 – 2268)	2092.5 (2052 – 2315.25)	NS
$ADC95p^2_{20\%}$	2551.5 (2301.75 – 2774.25)	2443.5 (2180.25 – 3051)	NS
$ADC95p^2_{40\%}$	2467.75 (2274.75 – 2619)	2187.5 (2055.5 – 2865.5)	NS
$ADC95p^2_{60\%}$	2312.5 (2036.25 – 2416.5)	1980 (1948.13 – 2376.75)	NS
$ADC95p^2_{80\%}$	2154.54 (1899.63 – 2341)	2052 (1944 – 2194.88)	NS
ADCsk [$\times 10^{-6}$ mm^2/s]			
$ADCsk^2_{5\%}$	0.71 (0.67 – 0.97)	0.62 (0.39 – 0.95)	NS
$ADCsk^2_{10\%}$	0.87 (0.69 – 1.06)	0.63 (0.4 – 1.13)	NS
$ADCsk^2_{20\%}$	1.15 (1.03 – 1.27)	1.19 (0.95 – 1.32)	NS
$ADCsk^2_{40\%}$	1.45 (1.34 – 1.53)	1.47 (1.3 – 1.48)	NS
$ADCsk^2_{60\%}$	1.76 (1.64 – 1.84)	1.7 (1.69 – 1.77)	NS
$ADCsk^2_{80\%}$	1.61 (1.45 – 1.86)	1.51 (1.47 – 1.6)	NS
ADCkurt [$\times 10^{-6}$ mm^2/s]			
$ADCkurt^2_{5\%}$	1.68 (1.2 – 2.15)	1.75 (0.94 – 2.75)	NS
$ADCkurt^2_{10\%}$	2.14 (1.46 – 2.45)	1.67 (1.38 – 2.99)	NS
$ADCkurt^2_{20\%}$	1.5 (1.18 – 1.69)	1.71 (1.56 – 1.78)	NS
$ADCkurt^2_{40\%}$	1.84 (1.52 – 2.28)	1.83 (1.57 – 2.55)	NS
$ADCkurt^2_{60\%}$	3.14 (2.37 – 4.04)	3.11 (2.81 – 3.77)	NS
$ADCkurt^2_{80\%}$	2.85 (1.8 – 3.74)	2.2 (2.19 – 2.89)	NS
ADCentr [$\times 10^{-6}$ mm^2/s]			
$ADCentr^2_{5\%}$	8.26 (8.2 – 8.28)	8.3 (8.24 – 8.31)	NS
$ADCentr^2_{10\%}$	8.32 (8.3 – 8.38)	8.31 (8.3 – 8.37)	NS
$ADCentr^2_{20\%}$	8.42 (8.39 – 8.44)	8.41 (8.31 – 8.52)	NS
$ADCentr^2_{40\%}$	8.23 (8.12 – 8.29)	8.14 (8.07 – 8.31)	NS
$ADCentr^2_{60\%}$	8.01 (7.92 – 8.1)	7.85 (7.85 – 8.04)	NS
$ADCentr^2_{80\%}$	7.96 (7.81 – 8.08)	7.96 (7.87 – 8.03)	NS

Table S7. Median values, Interquartile ranges (IQR) and results of Mann-Whitney U test of parameters for assessment of final response to treatment (evaluating percentage differences T2 vs T0, T1 vs T0 and T2 vs T1 parameters and using Lugano on T2 images).

**T2-T0 PARAMETERS TO ASSESS T2 RESPONSE**			
**Parameters**	**Median (IQR) - CMR**	**Median (IQR) - PMR**	**p**
ΔDV [%]			

$\Delta DV^{02}_{5\%}$	3.17 (0.91 – 12.18)	1.82 (0.92 – 17.72)	NS
$\Delta DV^{02}_{10\%}$	22.3 (8.11 – 35.54)	2.85 (-6.48 – 31.8)	NS
$\Delta DV^{02}_{20\%}$	30.64 (12.48 – 50.01)	15.12 (-8.69 – 16.82)	NS
$\Delta DV^{02}_{40\%}$	15.04 (4.49 – 34.57)	-10.05 (-22.72 – -1.69)	0.025
$\Delta DV^{02}_{60\%}$	7.47 (1.46 – 27.36)	-9.04 (-17.61 – 2)	NS
$\Delta DV^{02}_{80\%}$	3.03 (-4.98 – 14.8)	-9.27 (-15.68 – -0.01)	NS
**$\Delta ADCmean$ [%]**			
$\Delta ADCmean^{02}_{5\%}$	-4.84 (-7.22 – 4.7)	-1.07 (-2.08 – 8.16)	NS
$\Delta ADCmean^{02}_{10\%}$	-2.68 (-9.04 – 4.04)	-1.14 (-1.61 – 7.24)	NS
$\Delta ADCmean^{02}_{20\%}$	-7.53 (-12.12 – -3.2)	-3.3 (-13.58 – 1.46)	NS
$\Delta ADCmean^{02}_{40\%}$	-9.82 (-15 – -2.79)	-5.95 (-8.01 – -1.15)	NS
$\Delta ADCmean^{02}_{60\%}$	-4.43 (-8.48 – -1.48)	-7.58 (-9.6 – -2.13)	NS
$\Delta ADCmean^{02}_{80\%}$	-0.03 (-5.91 – 3.83)	-2.7 (-68.37 – 1.42)	NS
**$\Delta ADCsd$ [%]**			
$\Delta ADCsd^{02}_{5\%}$	-2.09 (-4.8 – 2.38)	4.6 (2.99 – 4.91)	0.039
$\Delta ADCsd^{02}_{10\%}$	-2.8 (-7.86 – -1.47)	3.41 (-3.24 – 8.4)	NS
$\Delta ADCsd^{02}_{20\%}$	1.09 (-5.24 – 3.5)	-0.77 (-12.97 – 5.39)	NS
$\Delta ADCsd^{02}_{40\%}$	1.26 (-9.9 – 11.88)	-8.76 (-23.17 – -8.74)	NS
$\Delta ADCsd^{02}_{60\%}$	-0.84 (-5.82 – 4.66)	-23.12 (-28.21 – -10.25)	0.025
$\Delta ADCsd^{02}_{80\%}$	-1.27 (-6.43 – 2.89)	-5.9 (-18.89 – 0.7)	NS
**$\Delta ADCmd$ [%]**			
$\Delta ADCmd^{02}_{5\%}$	-7.93 (-11.9 – 7.19)	0 (-1.65 – 14.81)	NS
$\Delta ADCmd^{02}_{10\%}$	-2.78 (-10.12 – 4.42)	0 (-1.55 – 13.79)	NS
$\Delta ADCmd^{02}_{20\%}$	-2.25 (-15.42 – 2.86)	2.35 (-15.68 – 5.53)	NS
$\Delta ADCmd^{02}_{40\%}$	-10.13 (-13.74 – -5.6)	2.47 (-5.03 – 6.78)	NS
$\Delta ADCmd^{02}_{60\%}$	-5.45 (-9.08 – -0.85)	0 (-1.03 – 0)	NS
$\Delta ADCmd^{02}_{80\%}$	-1.74 (-5.38 – 0.37)	0.81 (0.2 – 1.48)	NS
**$\Delta ADC5p$ [%]**			
$\Delta ADC5p^{02}_{5\%}$	21.05 (-2 – 50.15)	0 (-10.34 – 15.79)	NS
$\Delta ADC5p^{02}_{10\%}$	9.52 (-9.7 – 25.49)	-6.45 (-19.79 – 14.18)	NS
$\Delta ADC5p^{02}_{20\%}$	-12.77 (-24.7 – 0)	-17.78 (-26.4 – -12.14)	NS
$\Delta ADC5p^{02}_{40\%}$	-8.16 (-15.57 – -2.17)	14.13 (-10.89 – 15.07)	NS
$\Delta ADC5p^{02}_{60\%}$	-4.26 (-6.69 – 0.91)	4.26 (4.26 – 7.26)	0.014
$\Delta ADC5p^{02}_{80\%}$	0.41 (-1.03 – 3.51)	4.08 (-2.1 – 10.42)	NS
**$\Delta ADC95p$ [%]**			
$\Delta ADC95p^{02}_{5\%}$	-3.43 (-10.08 – 2.67)	0 (-1.02 – 2.01)	NS
$\Delta ADC95p^{02}_{10\%}$	-3.17 (-6.96 – -1.34)	-3.82 (-4.64 – 0.78)	NS
$\Delta ADC95p^{02}_{20\%}$	-3.08 (-8.52 – -1.81)	-8.28 (-16.74 – 4.72)	NS
$\Delta ADC95p^{02}_{40\%}$	-1.81 (-9.58 – 4.51)	-6.22 (-23.02 – -0.9)	NS
$\Delta ADC95p^{02}_{60\%}$	-2.28 (-6.13 – 1)	-16.45 (-20.01 – -6)	NS
$\Delta ADC95p^{02}_{80\%}$	-0.16 (-4.03 – 4.16)	-2.29 (-17.42 – 1.72)	NS
**$\Delta ADCsk$ [%]**			
$\Delta ADCsk^{02}_{5\%}$	5.62 (-7.99 – 47.59)	-8.75 (-49.67 – 30.46)	NS
$\Delta ADCsk^{02}_{10\%}$	8.2 (-4.54 – 33.58)	-19.46 (-57.36 – 15.52)	NS

$\Delta ADCsk^{02}_{20\%}$	13.97 (-4.55 – 28.26)	7.13 (-22.14 – 10.01)	NS
$\Delta ADCsk^{02}_{40\%}$	20.74 (-1.19 – 51.05)	-1.85 (-12.44 – 11.78)	NS
$\Delta ADCsk^{02}_{60\%}$	12.56 (-0.99 – 25.58)	-1.37 (-3.21 – 23.34)	NS
$\Delta ADCsk^{02}_{80\%}$	7.74 (1.07 – 20.81)	-0.85 (-5.19 – 2.11)	NS
ΔADCkurt [%]			
$\Delta ADCkurt^{02}_{5\%}$	1.32 (-13.02 – 38.33)	-21.06 (-57.5 – 55.29)	NS
$\Delta ADCkurt^{02}_{10\%}$	15.18 (-6.66 – 39.89)	-22.8 (-45.53 – 22.2)	NS
$\Delta ADCkurt^{02}_{20\%}$	3.33 (-32.93 – 88.24)	16.53 (6.17 – 30.37)	NS
$\Delta ADCkurt^{02}_{40\%}$	-7.09 (-71.11 – 179.08)	7.58 (-2.89 – 135.51)	NS
$\Delta ADCkurt^{02}_{60\%}$	36.4 (-15.42 – 62)	7.85 (-0.74 – 409.56)	NS
$\Delta ADCkurt^{02}_{80\%}$	10.44 (-19.39 – 50)	-0.92 (-11.81 – 48.97)	NS
ΔADCentr [%]			
$\Delta ADCentr^{02}_{5\%}$	0.03 (-0.5 – 0.52)	0.44 (-0.01 – 0.63)	NS
$\Delta ADCentr^{02}_{10\%}$	-0.14 (-0.54 – 0.27)	0.29 (-0.19 – 0.35)	NS
$\Delta ADCentr^{02}_{20\%}$	-0.57 (-1.04 – 0.39)	-0.68 (-0.82 – 0.49)	NS
$\Delta ADCentr^{02}_{40\%}$	-0.31 (-1.46 – 1.16)	-0.65 (-1.95 – -0.42)	NS
$\Delta ADCentr^{02}_{60\%}$	-0.57 (-1.33 – 0.22)	-1.33 (-2.75 – -1.01)	NS
$\Delta ADCentr^{02}_{80\%}$	-0.44 (-1.75 – 0.11)	-0.29 (-0.65 – -0.27)	NS
**T2-T1 PARAMETERS TO ASSESS T2 RESPONSE**			
ΔDV [%]			
$\Delta DV^{12}_{5\%}$	1.97 (-4.03 – 10.73)	5.99 (3.79 – 10.26)	NS
$\Delta DV^{12}_{10\%}$	6.1 (-6.93 – 29.1)	-3.71 (-8.13 – 25.81)	NS
$\Delta DV^{12}_{20\%}$	25.99 (3.96 – 34.99)	-3.27 (-12.07 – 8.5)	NS
$\Delta DV^{12}_{40\%}$	22.02 (6.47 – 35.58)	0.72 (-11.71 – 2.04)	NS
$\Delta DV^{12}_{60\%}$	10.8 (0.39 – 20.9)	1.8 (-3.01 – 5.44)	NS
$\Delta DV^{12}_{80\%}$	3.99 (-0.95 – 7.17)	-2.66 (-5.98 – 3.42)	NS
ΔADCmean [%]			
$\Delta ADCmean^{12}_{5\%}$	-0.08 (-1.93 – 4.95)	1.45 (-0.31 – 15.68)	NS
$\Delta ADCmean^{12}_{10\%}$	0.36 (-4.33 – 7.13)	0.23 (0.03 – 14.13)	NS
$\Delta ADCmean^{12}_{20\%}$	-2.04 (-4.11 – 2.02)	7.99 (-2.98 – 10.39)	NS
$\Delta ADCmean^{12}_{40\%}$	-3.96 (-6.86 – 2.47)	-0.17 (-3.56 – 7.54)	NS
$\Delta ADCmean^{12}_{60\%}$	-2.4 (-5.95 – 0.3)	-2.24 (-3.9 – 1.04)	NS
$\Delta ADCmean^{12}_{80\%}$	-0.71 (-1.26 – 0.98)	-2.16 (-67.49 – -1.2)	NS
ΔADCsd [%]			
$\Delta ADCsd^{12}_{5\%}$	-1.79 (-4.5 – 1.22)	6.35 (5.09 – 10.09)	0.017
$\Delta ADCsd^{12}_{10\%}$	-3.42 (-5.4 – -1.49)	3.65 (-3.57 – 16.55)	NS
$\Delta ADCsd^{12}_{20\%}$	-5.17 (-7.56 – 3.43)	-2.08 (-6.1 – 19.46)	NS
$\Delta ADCsd^{12}_{40\%}$	-1.23 (-2.34 – 1.94)	-5.99 (-6.62 – 10.69)	NS
$\Delta ADCsd^{12}_{60\%}$	-2.53 (-5.55 – 6.62)	-6.59 (-13.85 – 5.2)	NS
$\Delta ADCsd^{12}_{80\%}$	-0.02 (-3.56 – 5.72)	-2.56 (-3.68 – 2.72)	NS
ΔADCmd [%]			
$\Delta ADCmd^{12}_{5\%}$	-2.2 (-5.04 – 9.69)	2.3 (-1.04 – 22.57)	NS
$\Delta ADCmd^{12}_{10\%}$	-0.92 (-6.48 – 9.75)	2.2 (-1 – 25.87)	NS
$\Delta ADCmd^{12}_{20\%}$	-2.97 (-9.03 – 6.39)	0 (-4.35 – 14.81)	NS
$\Delta ADCmd^{12}_{40\%}$	-5.8 (-8.93 – 2.67)	2.47 (-3.08 – 6.78)	NS

$\Delta ADCmd^{12}_{60\%}$	-2.06 (-5.29 – 1.32)	-2.6 (-2.7 – 0.76)	NS
$\Delta ADCmd^{12}_{80\%}$	-0.21 (-1.96 – 0.85)	0 (0 – 1.56)	NS
$\boldsymbol{\Delta ADC5p}$ ***[%]***			
$\Delta ADC5p^{12}_{5\%}$	14.87 (0 – 43.42)	0 (-5.56 – 40)	NS
$\Delta ADC5p^{12}_{10\%}$	9.52 (6.45 – 18.01)	-12.12 (-21.21 – 23.44)	NS
$\Delta ADC5p^{12}_{20\%}$	14.24 (-13.29 – 39.29)	-10.26 (-11.66 – 33.44)	NS
$\Delta ADC5p^{12}_{40\%}$	2.04 (-4.35 – 13.41)	9.05 (-2.84 – 9.58)	NS
$\Delta ADC5p^{12}_{60\%}$	-2.29 (-8.05 – 0.15)	3.92 (0.98 – 4.62)	NS
$\Delta ADC5p^{12}_{80\%}$	0 (-0.52 – 0)	0 (-0.37 – 0)	NS
$\boldsymbol{\Delta ADC95p}$ ***[%]***			
$\Delta ADC95p^{12}_{5\%}$	-2.02 (-6.96 – 2.69)	1.4 (1.32 – 5.59)	NS
$\Delta ADC95p^{12}_{10\%}$	-3.17 (-4.94 – 0.7)	-1.31 (-2.21 – 8.16)	NS
$\Delta ADC95p^{12}_{20\%}$	-1.45 (-8.28 – 3.35)	4.62 (-3.39 – 28.27)	NS
$\Delta ADC95p^{12}_{40\%}$	-1.24 (-8.23 – 2.75)	-2.97 (-3.66 – 23.53)	NS
$\Delta ADC95p^{12}_{60\%}$	-1.51 (-4.92 – 1.63)	-3.73 (-8.31 – 7.53)	NS
$\Delta ADC95p^{12}_{80\%}$	0.49 (-1.52 – 1.97)	-2.45 (-3.16 – 2.16)	NS
$\boldsymbol{\Delta ADCsk}$ ***[%]***			
$\Delta ADCsk^{12}_{5\%}$	6.08 (-12.33 – 17.26)	2.84 (-43.62 – 80.25)	NS
$\Delta ADCsk^{12}_{10\%}$	5.68 (-7.22 – 19.65)	-23.59 (-55.15 – 69.1)	NS
$\Delta ADCsk^{12}_{20\%}$	10.62 (1.18 – 23.06)	9.47 (1.19 – 22.39)	NS
$\Delta ADCsk^{12}_{40\%}$	3.32 (-2.82 – 14.46)	13.27 (4.76 – 20.27)	NS
$\Delta ADCsk^{12}_{60\%}$	2.96 (-2.65 – 10.7)	14.56 (4.74 – 15.08)	NS
$\Delta ADCsk^{12}_{80\%}$	2.92 (-1.87 – 8.62)	2.23 (-3.95 – 4.77)	NS
$\boldsymbol{\Delta ADCkurt}$ ***[%]***			
$\Delta ADCkurt^{12}_{5\%}$	7.33 (-10.76 – 52.92)	-1.24 (-44.56 – 122)	NS
$\Delta ADCkurt^{12}_{10\%}$	16.03 (1.97 – 68.71)	-26.04 (-27 – 57.86)	NS
$\Delta ADCkurt^{12}_{20\%}$	59.4 (2.86 – 80.17)	19.05 (12.92 – 150.96)	NS
$\Delta ADCkurt^{12}_{40\%}$	34.63 (-9.25 – 92.23)	17.77 (8.5 – 152.04)	NS
$\Delta ADCkurt^{12}_{60\%}$	10.11 (-6.02 – 39.9)	24.49 (12.96 – 48.48)	NS
$\Delta ADCkurt^{12}_{80\%}$	5.59 (-20.62 – 17.68)	1.58 (-9.76 – 20.15)	NS
$\boldsymbol{\Delta ADCentr}$ ***[%]***			
$\Delta ADCentr^{12}_{5\%}$	-0.12 (-0.32 – 0.1)	0.33 (0.18 – 1.14)	NS
$\Delta ADCentr^{12}_{10\%}$	-0.25 (-0.5 – -0.02)	0.4 (-0.32 – 0.77)	NS
$\Delta ADCentr^{12}_{20\%}$	-0.52 (-1.38 – 0.29)	-0.2 (-0.61 – 1.24)	NS
$\Delta ADCentr^{12}_{40\%}$	0 (-0.59 – 0.39)	-0.8 (-0.81 – 0.51)	NS
$\Delta ADCentr^{12}_{60\%}$	-0.05 (-0.78 – 0.36)	-0.82 (-1.85 – -0.14)	NS
$\Delta ADCentr^{12}_{80\%}$	-0.24 (-0.53 – 0.49)	-0.24 (-0.53 – 0.33)	NS

S2. Boxplots and ROC curves for significant parameters

Prediction of final response to treatment

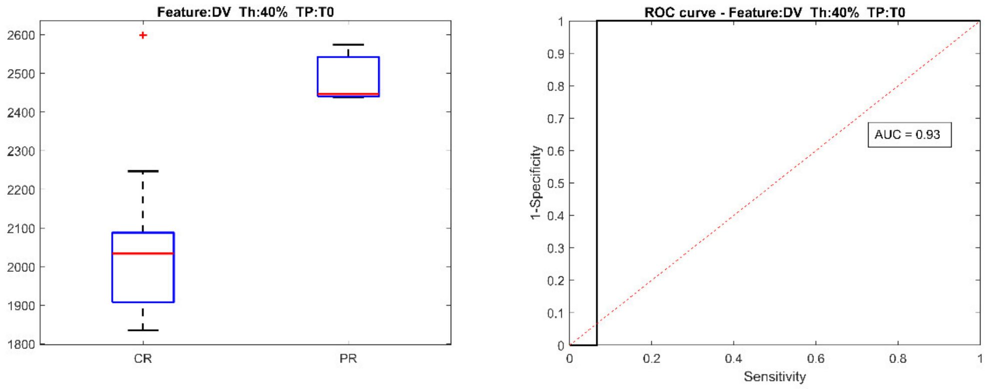

Figure S1. Boxplot (left) and ROC curve (right) for $DV_{40\%}^{0}$ for prediction of final response to treatment.

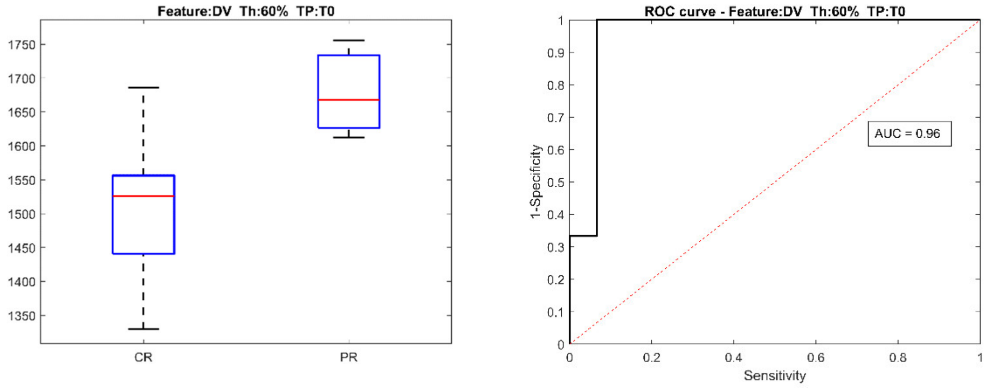

Figure S2. Boxplot (left) and ROC curve (right) for $DV_{60\%}^{0}$ for prediction of final response to treatment.

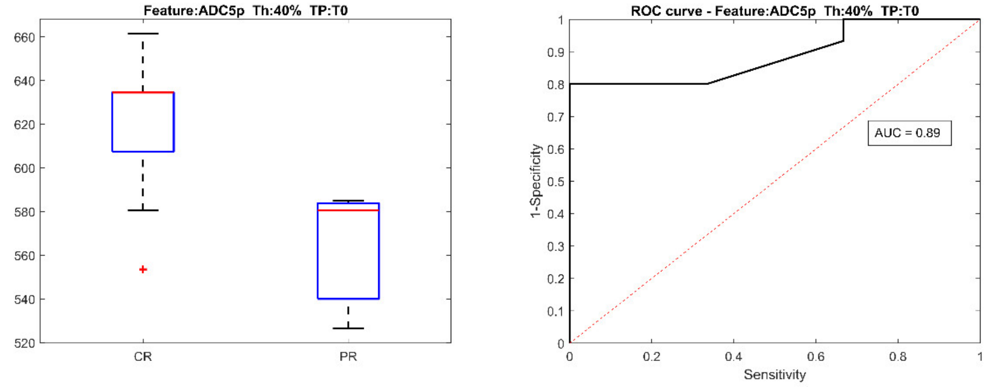

Figure S3. Boxplot (left) and ROC curve (right) for $ADC5p_{40\%}^{0}$ for prediction of final response to treatment.

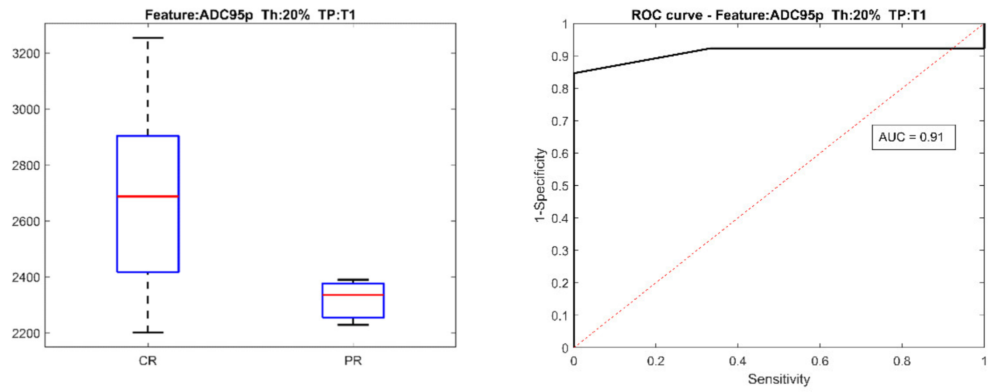

Figure S4. Boxplot (left) and ROC curve (right) for $ADC95p^{1}_{20\%}$ for prediction of final response to treatment.

Assessment of intermediate response to treatment

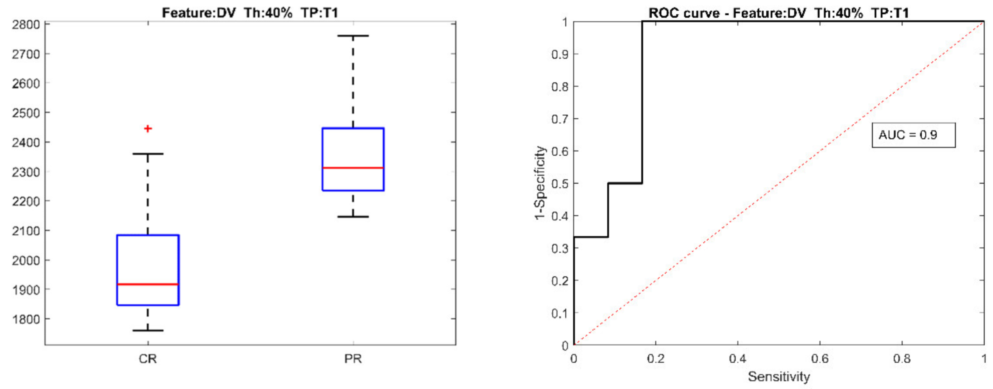

Figure S5. Boxplot (left) and ROC curve (right) for $DV^{1}_{40\%}$ for assessment of intermediate response to treatment.

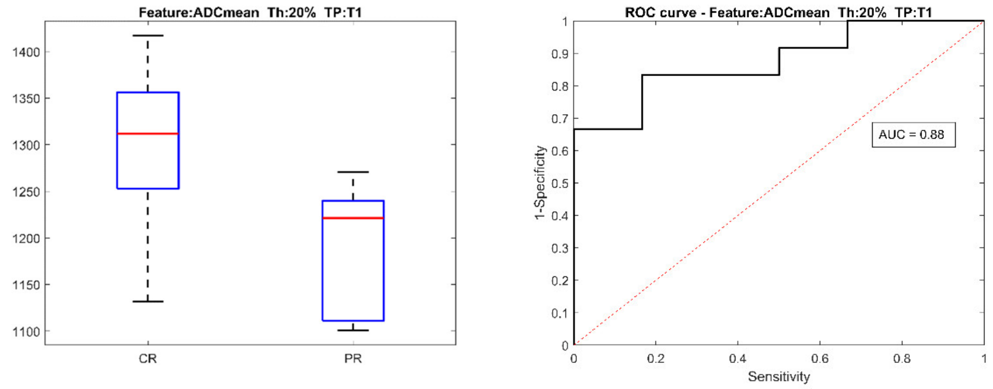

Figure S6. Boxplot (left) and ROC curve (right) for $ADCmean^{1}_{20\%}$ for assessment of intermediate response to treatment.

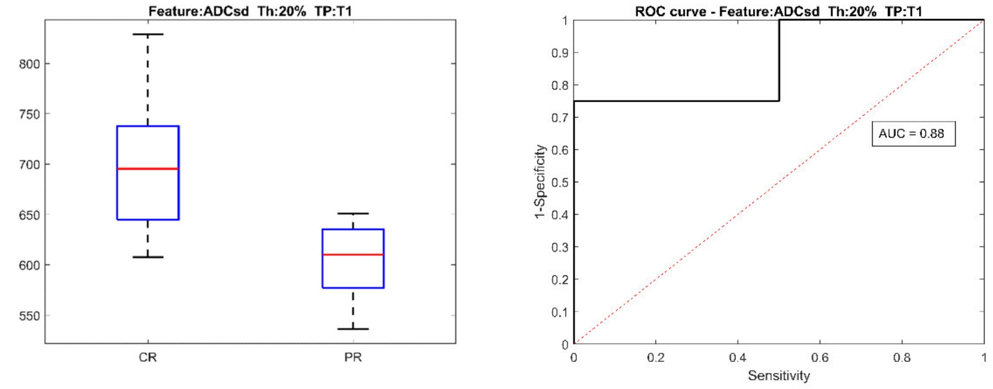

Figure S7. Boxplot (left) and ROC curve (right) for $ADCsd^{1}_{20\%}$ for assessment of intermediate response to treatment.

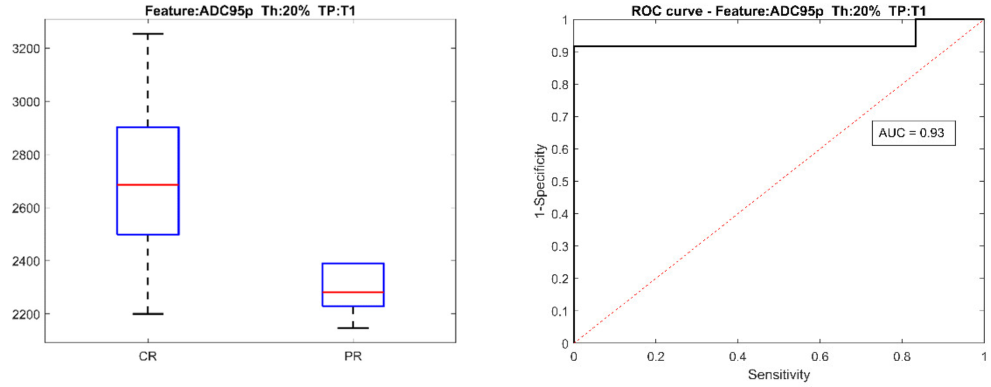

Figure S8. Boxplot (left) and ROC curve (right) for $ADC95p^{1}_{20\%}$ for assessment of intermediate response to treatment.

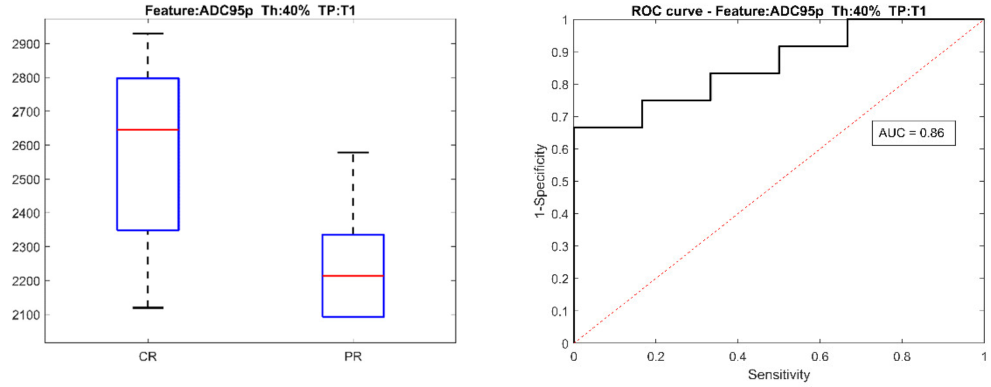

Figure S9. Boxplot (left) and ROC curve (right) for $ADC95p^{1}_{40\%}$ for assessment of intermediate response to treatment.

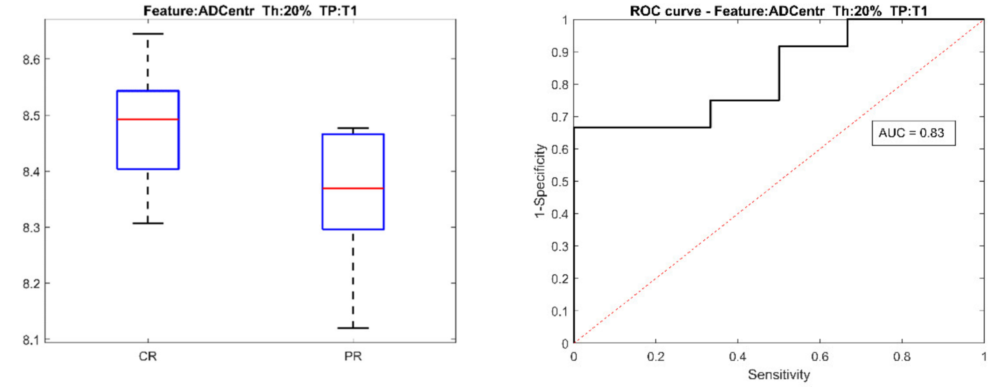

Figure S10. Boxplot (left) and ROC curve (right) for $ADCentr^{1}_{20\%}$ for assessment of intermediate response to treatment.

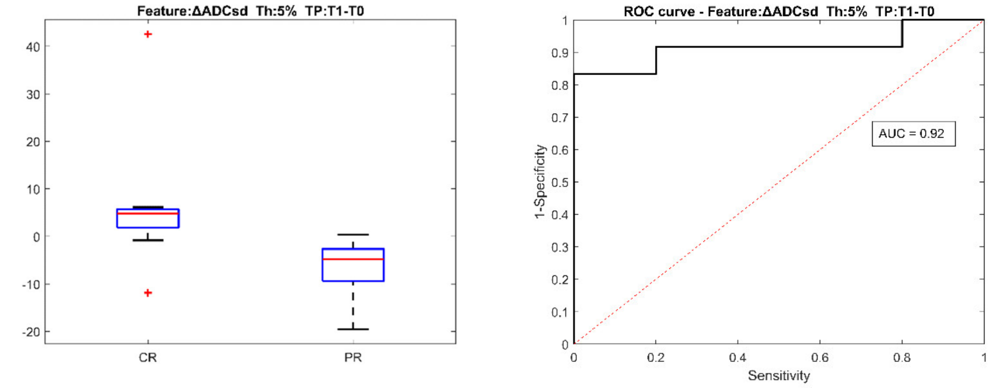

Figure S11. Boxplot (left) and ROC curve (right) for $\Delta ADCsd^{01}_{5\%}$ for assessment of intermediate response to treatment.

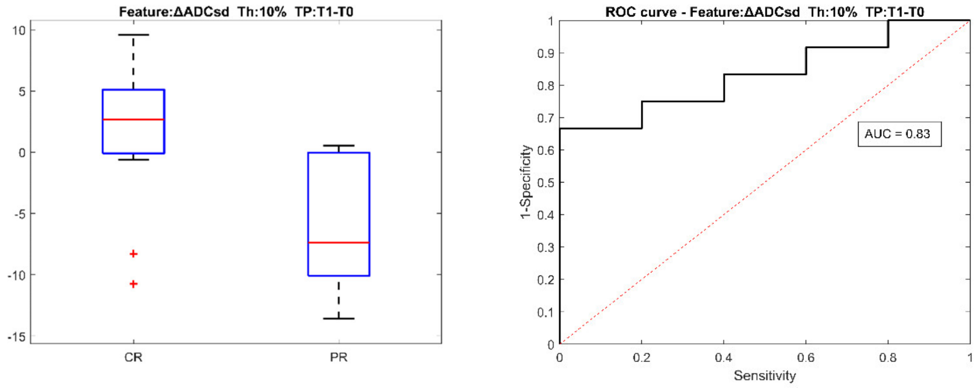

Figure S12. Boxplot (left) and ROC curve (right) for $\Delta ADCsd_{10\%}^{01}$ for assessment of intermediate response to treatment.

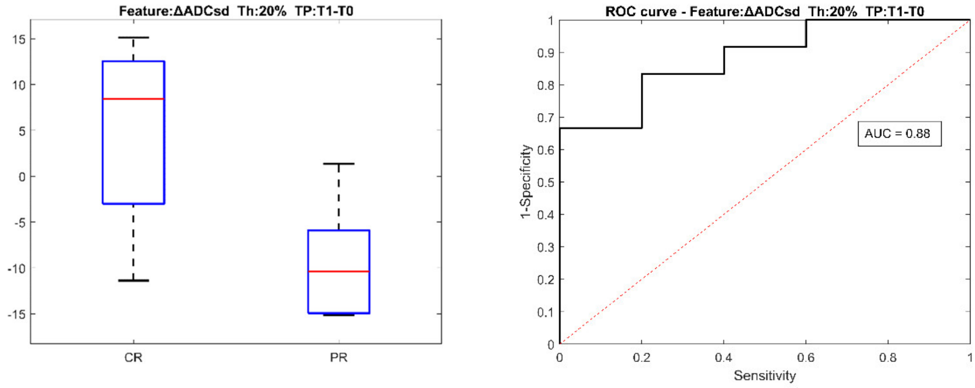

Figure S13. Boxplot (left) and ROC curve (right) for $\Delta ADCsd_{20\%}^{01}$ for assessment of intermediate response to treatment.

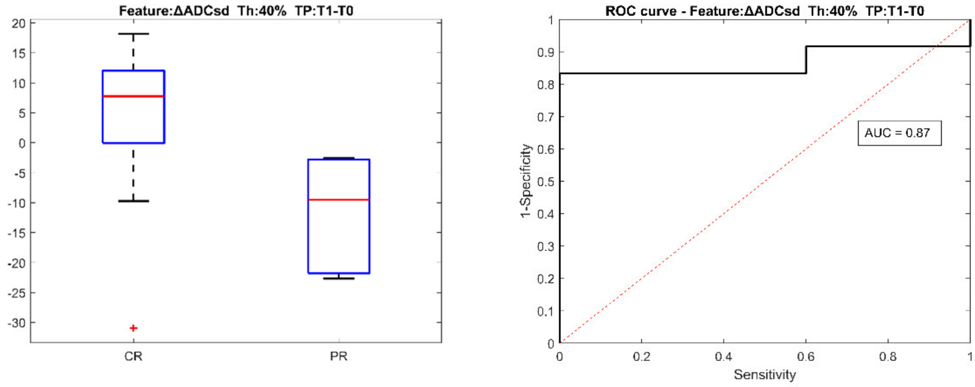

Figure S14. Boxplot (left) and ROC curve (right) for $\Delta ADCsd_{40\%}^{01}$ for assessment of intermediate response to treatment.

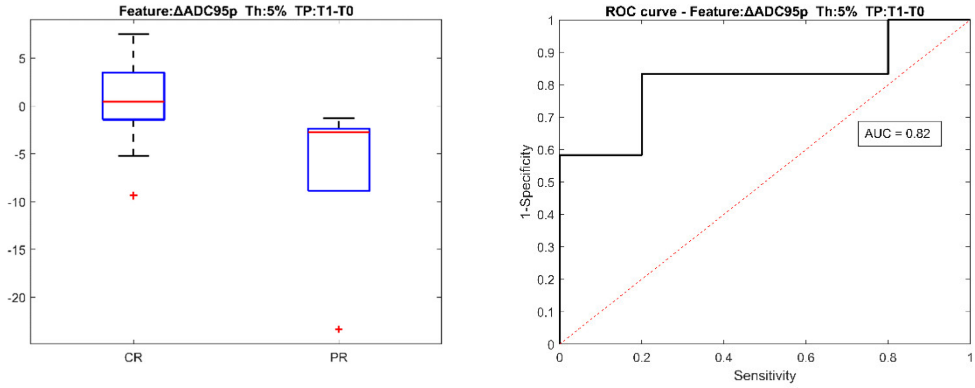

Figure S15. Boxplot (left) and ROC curve (right) for $\Delta ADC95p_{5\%}^{01}$ for assessment of intermediate response to treatment.

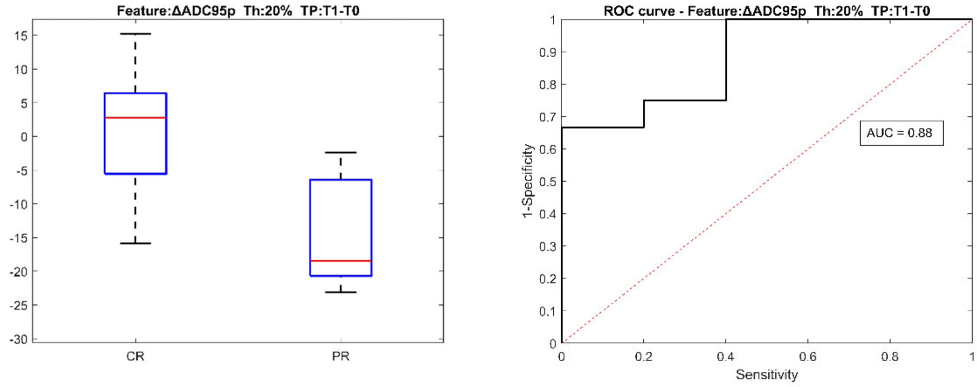

Figure S16. Boxplot (left) and ROC curve (right) for $\Delta ADC95p_{20\%}^{01}$ for assessment of intermediate response to treatment.

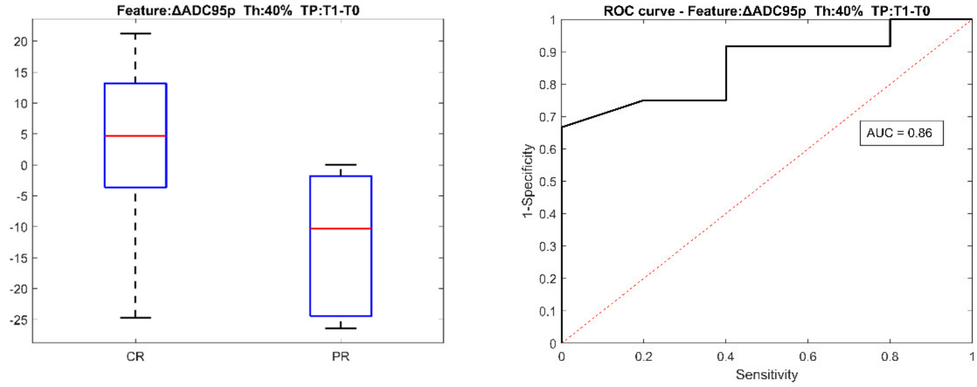

Figure S17. Boxplot (left) and ROC curve (right) for $\Delta ADC95p_{40\%}^{01}$ for assessment of intermediate response to treatment.

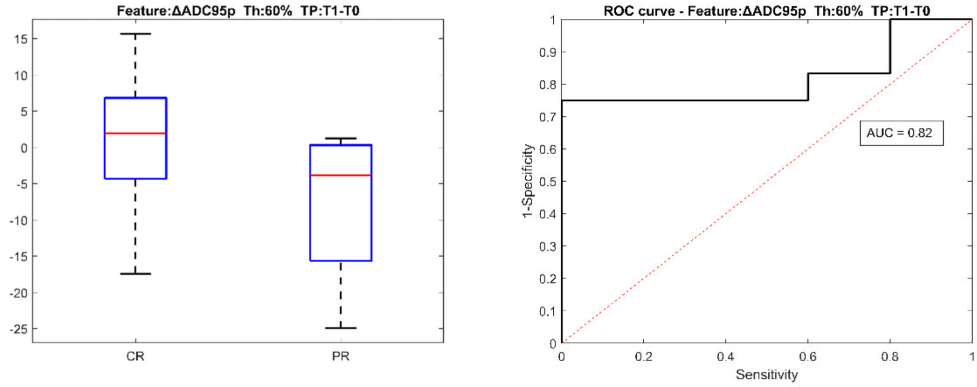

Figure S18. Boxplot (left) and ROC curve (right) for $\Delta ADC95p_{60\%}^{01}$ for assessment of intermediate response to treatment.

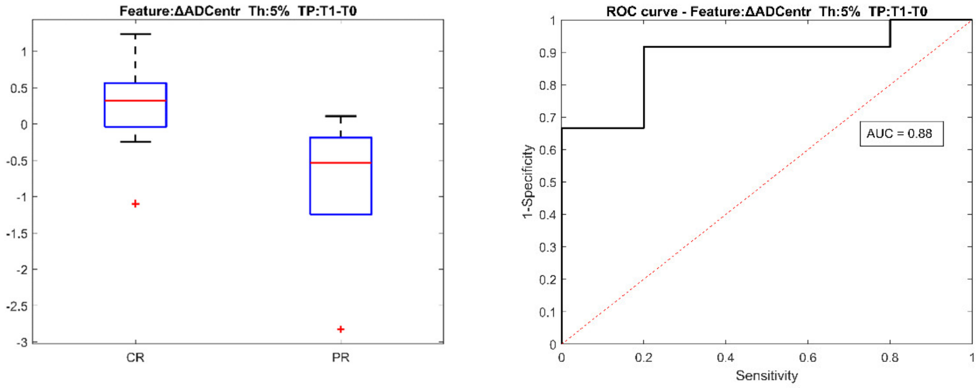

Figure S19. Boxplot (left) and ROC curve (right) for $\Delta ADCentr_{5\%}^{01}$ for assessment of intermediate response to treatment.

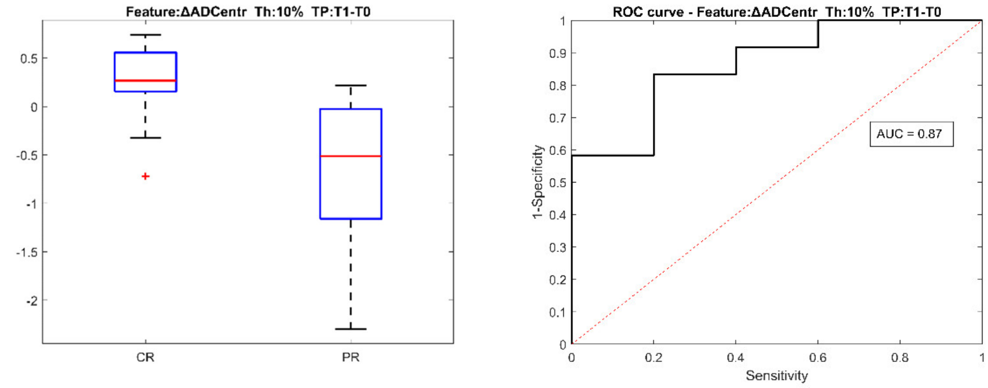

Figure S20. Boxplot (left) and ROC curve (right) for $\Delta ADCentr_{10\%}^{01}$ for assessment of intermediate response to treatment.

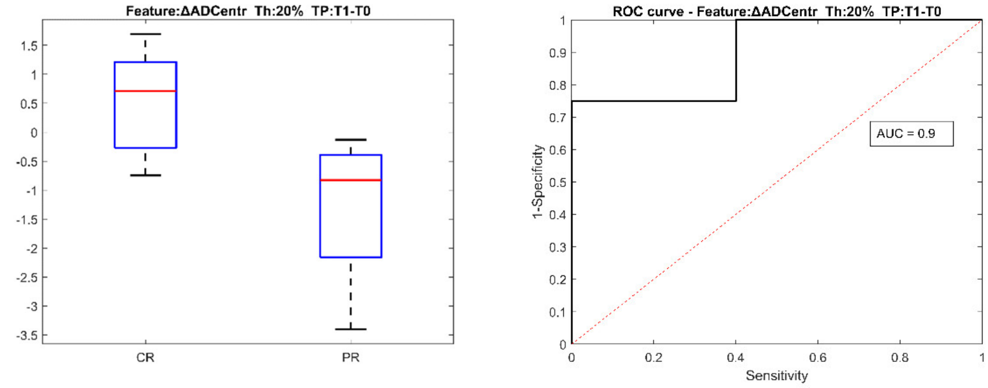

Figure S21. Boxplot (left) and ROC curve (right) for $\Delta ADCentr_{20\%}^{01}$ for assessment of intermediate response to treatment.

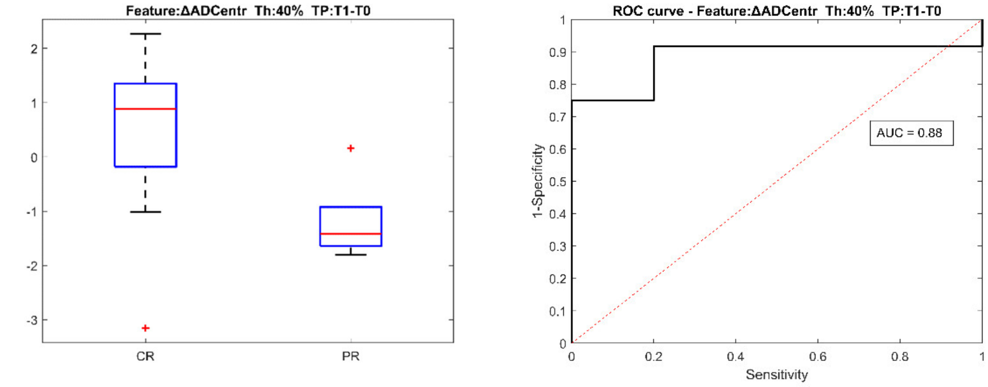

Figure S22. Boxplot (left) and ROC curve (right) for $\Delta ADCentr_{40\%}^{01}$ for assessment of intermediate response to treatment.

Assessment of final response to treatment

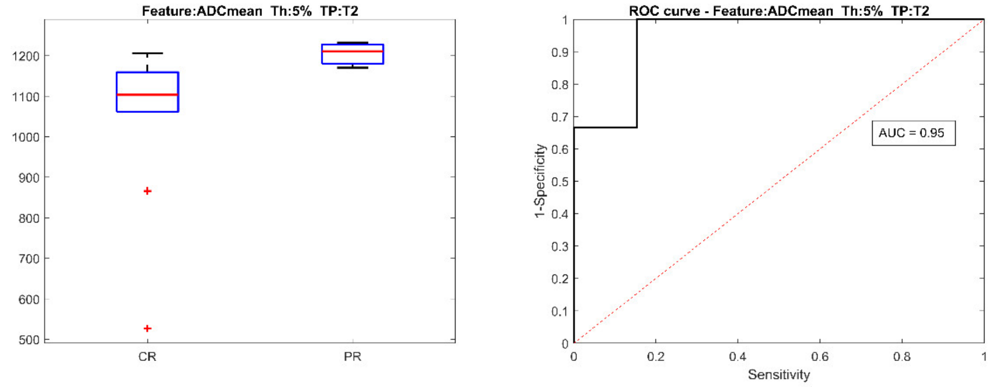

Figure S23. Boxplot (left) and ROC curve (right) for $ADCmean_{5\%}^{2}$ for assessment of final response to treatment.

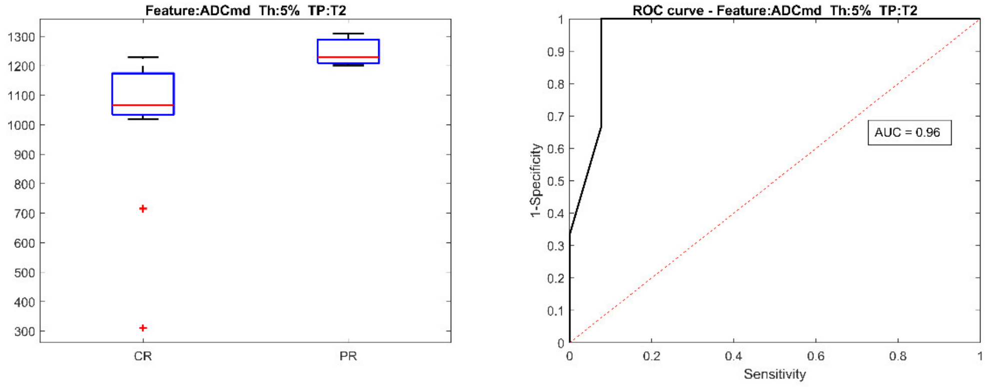

Figure S24. Boxplot (left) and ROC curve (right) for $ADCmd^{2}_{5\%}$ for assessment of final response to treatment.

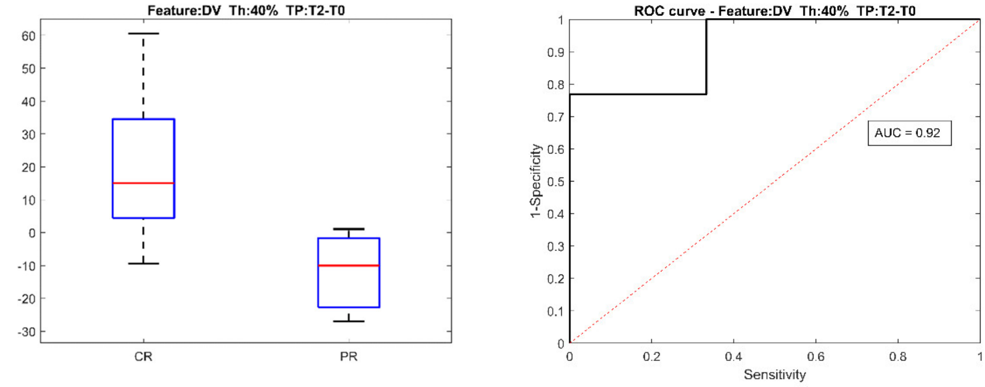

Figure S25. Boxplot (left) and ROC curve (right) for $\Delta DV^{02}_{40\%}$ for assessment of final response to treatment.

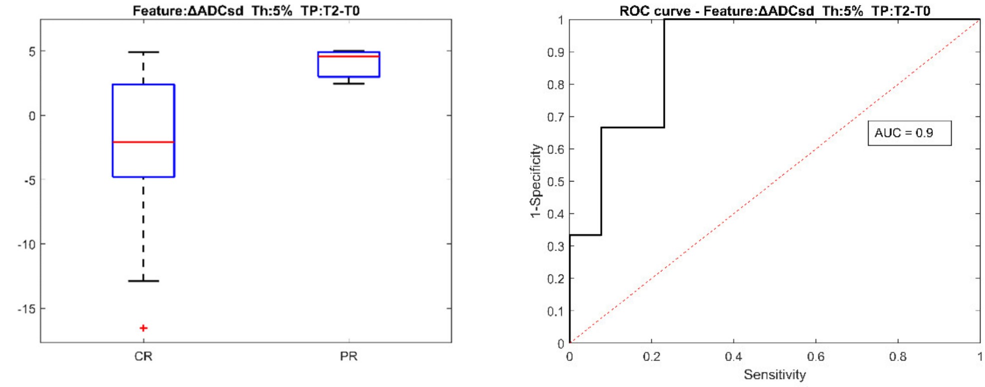

Figure S26. Boxplot (left) and ROC curve (right) for $\Delta ADCsd^{02}_{5\%}$ for assessment of final response to treatment.

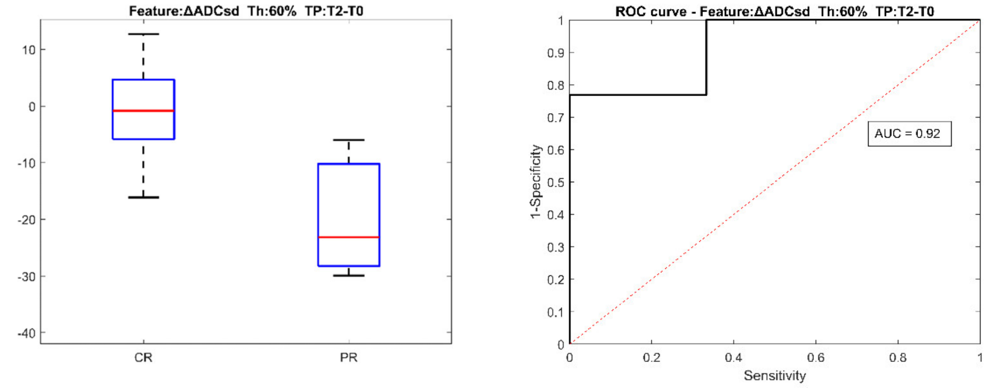

Figure S27. Boxplot (left) and ROC curve (right) for $\Delta ADCsd^{02}_{60\%}$ for assessment of final response to treatment.

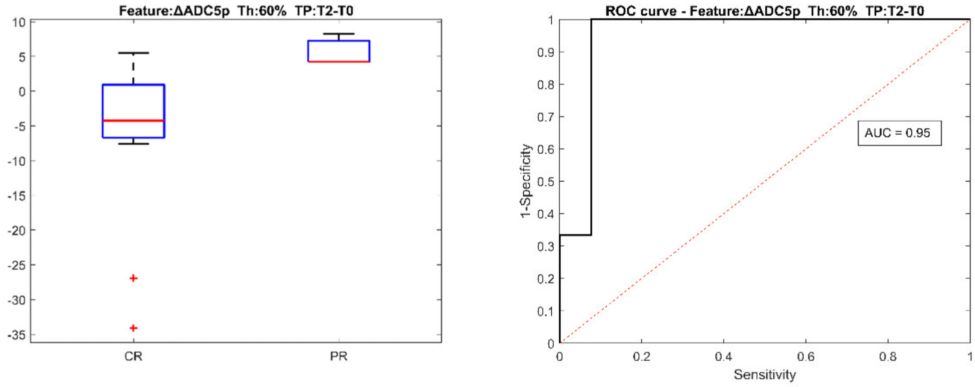

Figure S28. Boxplot (left) and ROC curve (right) for $\Delta ADC5p_{60\%}^{02}$ for assessment of final response to treatment.

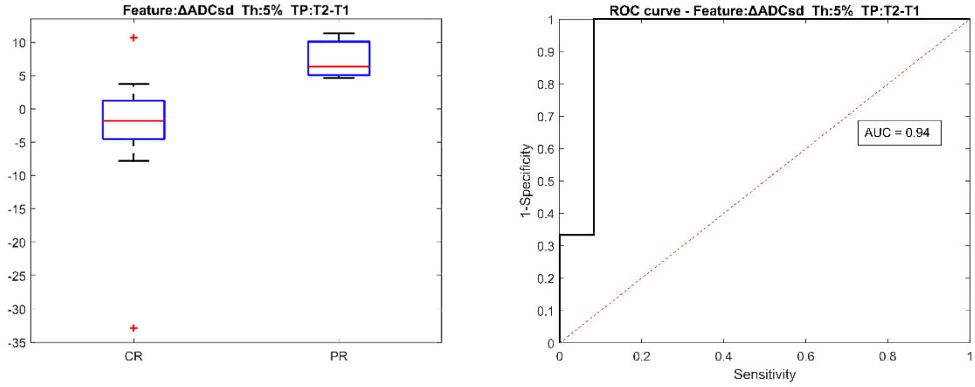

Figure S29. Boxplot (left) and ROC curve (right) for $\Delta ADCsd_{5\%}^{12}$ for assessment of final response to treatment.
